# Repeated evolution of circadian clock dysregulation in cavefish populations

**DOI:** 10.1371/journal.pgen.1009642

**Published:** 2021-07-12

**Authors:** Katya L. Mack, James B. Jaggard, Jenna L. Persons, Emma Y. Roback, Courtney N. Passow, Bethany A. Stanhope, Estephany Ferrufino, Dai Tsuchiya, Sarah E. Smith, Brian D. Slaughter, Johanna Kowalko, Nicolas Rohner, Alex C. Keene, Suzanne E. McGaugh

**Affiliations:** 1 Biology, Stanford University, Stanford, California, United States of America; 2 Department of Biological Sciences, Florida Atlantic University, Jupiter, Florida, United States of America; 3 Center for Sleep Sciences and Medicine, Department of Psychiatry and Behavioral Sciences, Stanford University, Stanford, California, United States of America; 4 Stowers Institute for Medical Research, Kansas City, Missouri, United States of America; 5 Ecology, Evolution, and Behavior, University of Minnesota, Saint Paul, Minnesota, United States of America; 6 Wilkes Honors College, Florida Atlantic University, Jupiter, Florida, United States of America; 7 Department of Molecular and Integrative Physiology, The University of Kansas Medical Center, Kansas City, Kansas, United States of America; University of Rochester, UNITED STATES

## Abstract

Circadian rhythms are nearly ubiquitous throughout nature, suggesting they are critical for survival in diverse environments. Organisms inhabiting largely arrhythmic environments, such as caves, offer a unique opportunity to study the evolution of circadian rhythms in response to changing ecological pressures. Populations of the Mexican tetra, *Astyanax mexicanus*, have repeatedly invaded caves from surface rivers, where individuals must contend with perpetual darkness, reduced food availability, and limited fluctuations in daily environmental cues. To investigate the molecular basis for evolved changes in circadian rhythms, we investigated rhythmic transcription across multiple independently-evolved cavefish populations. Our findings reveal that evolution in a cave environment has led to the repeated disruption of the endogenous biological clock, and its entrainment by light. The circadian transcriptome shows widespread reductions and losses of rhythmic transcription and changes to the timing of the activation/repression of core-transcriptional clock. In addition to dysregulation of the core clock, we find that rhythmic transcription of the melatonin regulator *aanat2* and melatonin rhythms are disrupted in cavefish under darkness. Mutants of *aanat2* and core clock gene *rorca* disrupt diurnal regulation of sleep in *A*. *mexicanus*, phenocopying circadian modulation of sleep and activity phenotypes of cave populations. Together, these findings reveal multiple independent mechanisms for loss of circadian rhythms in cavefish populations and provide a platform for studying how evolved changes in the biological clock can contribute to variation in sleep and circadian behavior.

## Author summary

Biological rhythms are molecular, physiological, and behavioral changes that follow a daily cycle and allow for animals to coordinate critical biological processes with their external environment. While these clocks are ubiquitous from unicellular life through humans, little is known about how they evolve in the absence of daily cycling within an environment. In this study, we sought to understand the evolutionary response of the biological clock when organisms become established in an environment that lacks daily fluctuations in light, temperature, and other environmental factors. *Astyanax mexicanus* have repeatedly moved from surface rivers into caves where they live in complete darkness. We find that multiple populations of cavefish have disrupted biological clocks compared to their surface relatives, but that these clocks are disrupted via different molecular mechanisms in different populations. Our results suggest that changes to the biological clock in these populations may also affect aspects of cavefish behavior, like the sleep-wake cycle. This study demonstrates that moving into an environment without daily cycles has led to predictable disruptions to the biological clock among cavefish populations, but that the clock itself can be broken multiple ways.

## Introduction

Circadian rhythms that maintain 24-hour oscillations in physiology and behavior are nearly ubiquitous in nature [1,2]. Considered an adaptive mechanism for organisms to anticipate predictable changes in their environment [3,4], the biological clock coordinates diverse biological processes, from the sleep-wake cycle and metabolism in animals, to growth and photosynthesis in plants [5–7]. In vertebrates, circadian rhythms are regulated by transcriptional feedback loops, where clock proteins directly or indirectly regulate the expression of the genes from which they are transcribed. The feedback loops of the circadian clock result in oscillations of gene expression of ~24 hours [8]. These oscillating transcripts make up the circadian transcriptome and are a substantial source of rhythmic physiology and behavior [9–11]. While the biological clock is endogenous, environmental time-cues (“zeitgebers”) including light, temperature, and food availability, synchronize the clock with an organism’s external environment (e.g., entrainment) [12–15]. Subjecting animals to light-dark cycles that differ from that of a 24-hour day has profound impacts on organismal health, including reduced performance, increased illness, and decreased longevity [16–18]. However, despite a detailed understanding of the neural and molecular basis for circadian rhythms, less is known about the mechanisms underlying the evolution of circadian rhythms in response to changing ecological pressures.

When species become established in environments that are isolated from day-night cycles, the biological clock is predicted to become dispensable and eventually be lost altogether [19,20]. While this prediction seems intuitive, the deep evolutionary origins of the biological clock and its role in coordinating a wide diversity of biological processes also suggest strong adaptive constraint [20]. Consequently, systems that have moved to environments without zeitgebers, such as caves, provide a unique opportunity to understand the relationship between species’ ecology, biological clock evolution, and downstream clock-regulated processes [21–23]. While there is now substantial evidence for subterranean species with altered behavioral or physiological rhythms compared to surface relatives [20,21], only a few studies have focused on the molecular nature of circadian clocks in arrhythmic environments [22–24]. Further, no study so far has investigated genome-wide patterns of expression or sequence divergence associated with changes to circadian rhythms in any subterranean system.

The Mexican tetra, *Astyanax mexicanus*, exists as surface populations that live in rivers with robust light and temperature rhythms, and at least 30 cave populations that live in perpetual darkness with limited fluctuations in temperature or other environmental cues [25]. Cave populations of *A*. *mexicanus* have repeatedly evolved a suite of traits in cave environments, including degenerate eyes [26–28], reduced pigmentation [29–32], and changes in metabolism and behavior [33–42]. There is also now abundant evidence that circadian rhythms and sleep behavior are substantially altered in cavefish populations [24,34,43,44]. While cavefish largely maintain locomotor and physiological rhythms in light-dark conditions, multiple populations show loss of these rhythms under constant darkness [24,43–47]. Consistent with the notion that cave colonization is associated with molecular disruptions to circadian rhythms, an examination of the expression of the circadian clock gene *per1a* found that while this gene cycles in cave populations under laboratory conditions, transcriptional oscillations were dampened under dark-dark conditions and absent in cavefish collected from the wild [24]. Further, changes to circadian rhythms in cavefish have been found to reduce metabolic rate in constant darkness, suggesting changes to circadian biology may be advantageous under certain environmental conditions [43]. Indeed, cavefish appear to be resilient to many aspects of metabolic dysregulation including a diabetes-like phenotype, obesity, and sleep loss, all of which are linked to circadian dysregulation in humans [34,40,48,49].

In light of the strong evidence for divergence of behavioral and molecular rhythms between *A*. *mexicanus* surface and cave populations, the existence of multiple cave populations of independent origin provides a uniquely powerful comparative framework for studying the evolution of the circadian clock in this system. The repeated invasion and establishment of *A*. *mexicanus* in caves allows us to not only ask how the molecular underpinning of circadian rhythms are altered in cave populations, but also whether alterations to biological timekeeping are predictable and repeatable across populations. Here we use a combination of RNA-sequencing, population genomics, and RNA fluorescence *in situ* hybridization (FISH), and CRISPR/Cas9 induced mutations to characterize divergence in the circadian transcriptome and circadian function between three cave populations and one surface population. Altogether, our data demonstrates that the biological clock, a highly conserved mechanism across most metazoans, has been repeatedly and independently disrupted at the molecular level in unique origins of cavefish. Unique disruptions to circadian rhythms across different origins of cavefish provide a powerful platform for studying the relationship between naturally occurring clock mutants and circadian biology.

## Results

### Fewer genes show evidence of daily cycling in cavefish populations

To identify changes in rhythmic expression between cave and surface populations, we performed RNAseq with total RNA from whole animals in three cave populations (Molino, Pachón, and Tinaja) and one surface population (Río Choy) collected every 4-hours for one daily cycle under constant darkness at 30 days post-fertilization (dpf). Pachón and Tinaja are two populations representing what is considered the “old” lineage of *A*. *mexicanus* and are sister taxa, where Río Choy and Molino are considered “new” lineage fish and more closely related to one another than the old lineage populations [50]. Fish were raised in a light-dark cycle (14:10) in order to synchronize behavioral and molecular clocks and then transferred into constant darkness 24 hours prior to the start of the sampling period. An average of 14,197,772 reads were mapped per sample (S1 Data and Figs A-D in S1 Text), with 6 replicates per population collected at 6 timepoints (144 samples total). Filtering of genes with low expression (< 100 total counts across all samples) resulted in 21,048 annotated genes used for downstream analysis. Although the cave populations are not monophyletic [50], principle component analysis showed that the primary axis of differentiation among samples is habitat (*i*.*e*., cave or surface; Fig E in S1 Text).

**Fig 1:**
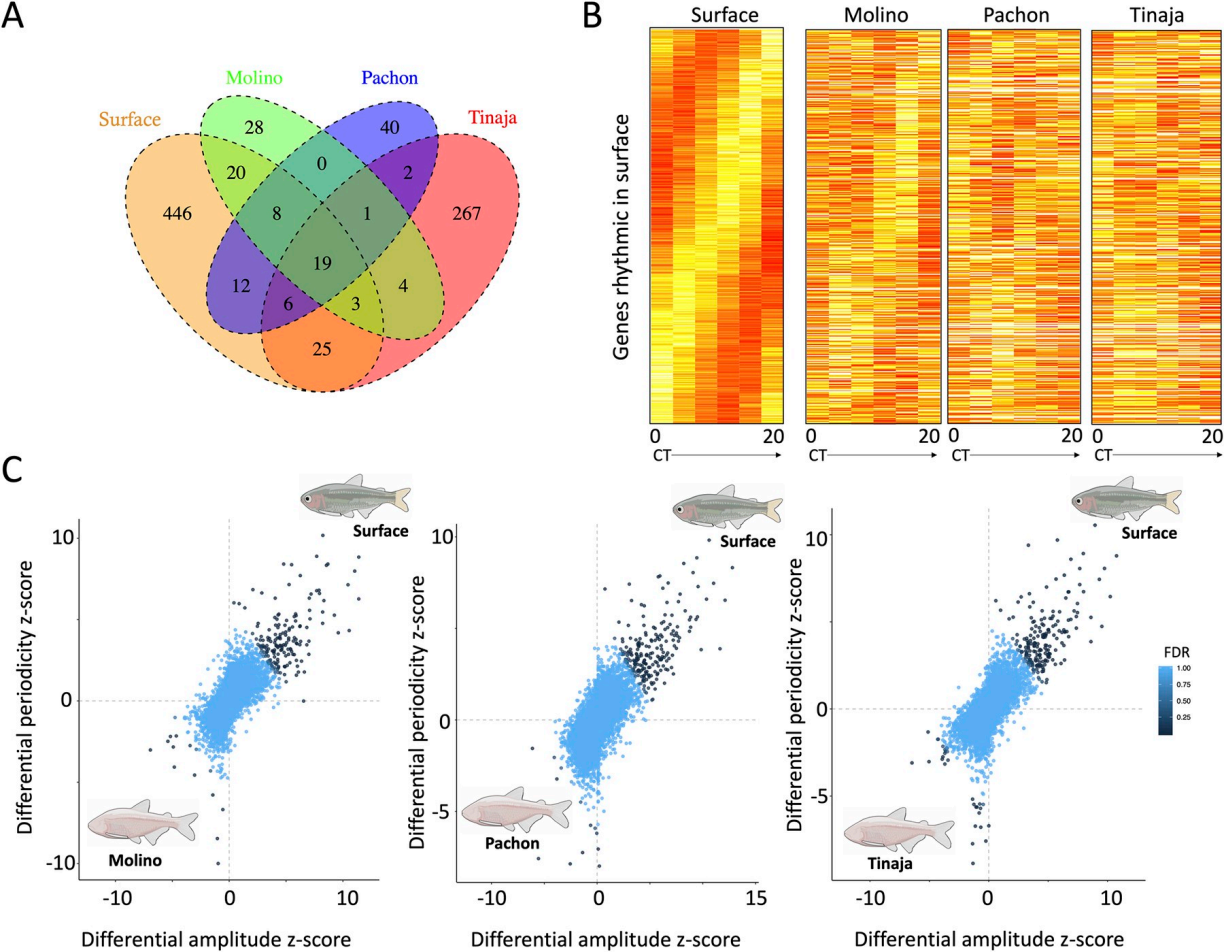
A. Overlap of genes with rhythmic expression between populations. B. Heatmap of genes with rhythmic patterns in surface fish (Río Choy), ordered by gene phase, compared to expression in cave populations. Each column represents gene expression at a single-time-point, sampled every four hours from 0–20 hours. Redder boxes correspond to higher expression. C. Identifying genes with changes in rhythmicity between cave and surface populations. Genes with greater amplitude values have larger differences between their expression peak and trough, where genes with greater periodicity show stronger cyclical oscillation patterns (see Methods). Genes are colored based on their *S*_DR_ *q*-values. Genes with positive values for both show increased rhythmicity in the surface population, where genes with negative values show increased rhythmicity in cave populations.

We used JTK_cycle, a non-parametric algorithm that detects cycling in genome-scale data sets, to detect 24-hour oscillations in transcript abundance [51]. We found that the surface population had the greatest number of rhythmic transcripts (539), followed by Tinaja (327), Pachón (88), and Molino (83), respectively (FDR < 0.05, see Table A in S1 Text), consistent with the notion that molecular cycling of gene expression is reduced in cavefish populations. Surprisingly few genes (19) showed significant cycling across all cave and surface populations (Fig 1A and 1B). Genes with rhythmic transcription in all four populations include genes that play known roles in circadian rhythm (e.g., *per1a*, *per1b*, *cipca*, *ciarta*, *dbpb*), suggesting that some components of the core-clock remain functional across cave fish populations (see S1 Data). In all populations except Molino, we found significant overlap between rhythmic transcripts in *A*. *mexicanus* and those identified in zebrafish [52,53] (See Methods; at *p* < 0.05: surface, Pachón and Tinaja, all *p* < 1 x 10^−4^; Molino *p* = 1, hypergeometric tests). Rhythmic transcripts in both the surface and cave populations were enriched for the GO terms related to circadian rhythm, including “regulation of circadian rhythm” (GO:0042752)(surface, *q* = 4.85 x 10^−10^; Tinaja, *q* = 4.16 x 10^−8^; Pachón, *q* = 2.33 x 10^−6^; Molino, *q* = 4.98 x 10^−6^). Rhythmic transcripts in the surface population were also strongly enriched for “visual perception” (GO:0007601)(*q* = 4.80 x 10^−12^), “sensory perception of light stimulus” (GO:0007602) (*q* = 9.28 x 10^−12^), and “phototransduction” (GO:0007602)(*q* = 1.14 x 10^−7^), unlike cave populations (lists of enriched terms in S1 Data). The Tinaja cave population showed a surprisingly high number of uniquely rhythmic transcripts (267 transcripts cycling only in Tinaja). These transcripts were enriched for terms related to DNA-replication (GO:0006260)(*q* = 9.55 x 10^−4^), mitotic cell cycle (GO:0000278)(*q* = 4.48 x10^-3^), and DNA repair (GO:0006281)(*q* = 0.012) (S1 Data).

Highlighting that the loss of rhythmicity has evolved repeatedly among independent origins of the cave phenotype, losses of rhythmic expression were often population-specific. Where nearly 22% (117) of genes found to be rhythmic in the surface population were arrhythmic (*p-*value > 0.5) in all three cave populations, 77% (416) were arrhythmic in at least one cave population (Table A in S1 Text, S1 Data). Conversely, no genes that were rhythmic across all caves were arrhythmic in the surface population, suggesting that the loss of rhythmic expression is due to inhabiting a cave environment. Genes arrhythmic across all cave populations were enriched for GO categories including “sensory perception of light stimulus” (*q* = 1.48 x10^-6^) and “nervous system process” (*q =* 1.09 x 10^−5^) (full list of terms in S1 Data) and include genes involved in the regulation of circadian rhythm (Table C in S1 Text). For example, *arntl2* encodes a transcriptional activator that forms a core component of the circadian clock and shows conserved rhythmic activity in zebrafish [52] and mice [65]. Despite exhibiting a conserved function across vertebrates, *arntl2* is arrhythmic in all three cave populations (surface, *p* = 0.0004, *q* = 0.02; all cave populations, *p* > 0.5). Further, we found that surface transcripts were significantly more rhythmic in their expression (e.g., have lower *p-*values) than cave populations (Wilcoxon signed-rank test, *p* < 0.002 in all cave-surface pairwise comparisons) when comparing the distribution of JTK_cycle *p-*values for orthologs with annotated roles in circadian rhythm (61 genes, see Methods, S1 Data). Taken together, these findings reveal the loss of cycling in different core-clock genes across multiple, independently-derived cavefish populations.

### Cave and surface populations show differences in periodicity and amplitude of rhythmic transcripts

To identify genes with changes in rhythmicity between cave and surface populations, we calculated a differential rhythmicity score (*S*_DR_) for each gene for each cave-surface population pair [66]. This metric accounts for both changes in how rhythmic a transcript is, as defined by differences in the JTK_cycle *p*-value for each gene between surface and cave populations, as well as differences in the robustness of a transcript’s oscillation, as defined by differences in amplitude of gene expression between the cave and surface populations. We found that 103 genes showed greater rhythmicity in the surface fish for all surface-cave fish comparisons (e.g., surface-Pachón, surface-Molino, surface-Tinaja; S1 Data)(Fig 1C). This set of genes was highly enriched for the pathway “circadian clock system,” with a 21-fold enrichment compared to the background set of genes used in this analysis (*q* = 9.96 x 10^−11^), and was the only pathway significantly enriched after false-discovery rate correction. However, many alterations in rhythmicity appear to be cave population-specific: of the 251 genes for which we identified rhythmic changes between a cave and surface pair, ~60% were identified in only one or two population comparisons. Relatively few genes showed significantly improved rhythmicity in individual cave populations compared to surface fish (Fig 1C, genes below zero for differential periodicity and differential amplitude z-score), and no genes showed increases in rhythmicity across multiple cave populations.

**Fig 2:**
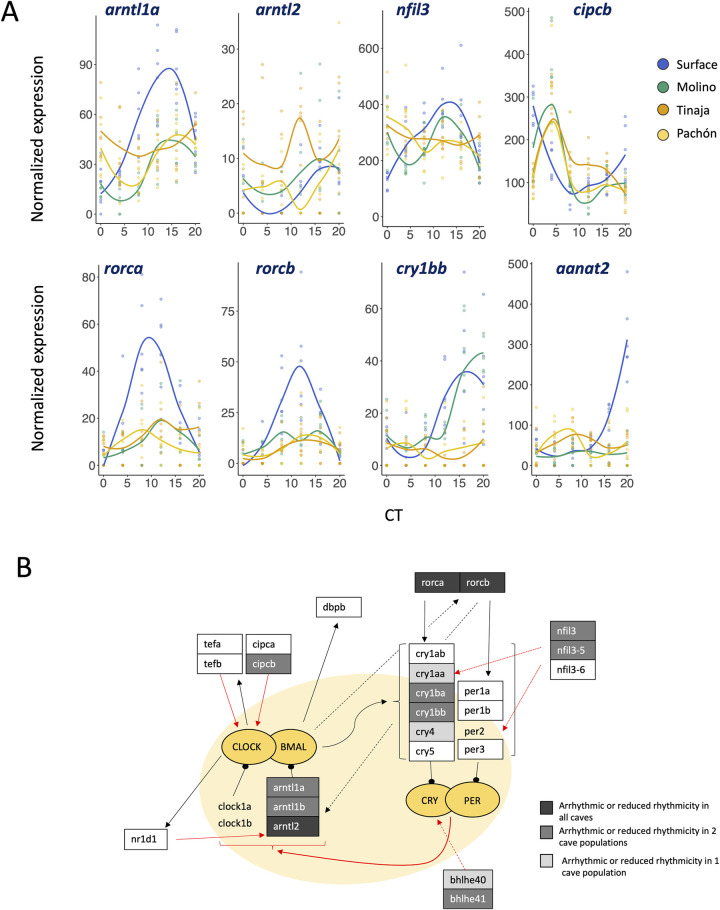
A. Key circadian genes with changes in rhythmicity in cave populations (see Fig U in S1 Text for all core circadian genes with changes in rhythmicity). Colored lines represent a loess regression of gene expression through time for each population. B. Simplified schematic of the circadian feedback loops based on proposed interactions in zebrafish[9,55]. Grey boxes indicate genes that are either arrhythmic or show significantly reduced rhythmicity between cave and surface. White boxed genes do not show significant differences between cave and surface. Highlighted in yellow is the core loop. Bright yellow circles represent regulating protein complexes. Red lines indicate negative regulation, black lines indicate positive regulation. Notably, *cry4* does not repress Clock/Bmal activation in zebrafish and may play a photoreceptor function[9,55]. Genes that are not boxed did not show evidence of rhythmic expression in any cave or surface population. Dotted lines are for visual clarity. Genes without an annotated ortholog in cavefish were not included in the schematic.

Genes with dampened rhythmic expression in cave populations include several primary and accessory components of the core transcriptional clock (Fig 2A)(Table 1). In the circadian clock’s primary feedback loop, members of the (bHLH)-PAS family (e.g., CLOCKs, ARNTLs) heterodimerize and bind to E-box DNA response elements to transcriptionally activate key clock proteins (e.g., PERs, CRYs). Negative feedback is then conferred by CRY:PER heterodimers which inhibit the CLOCK:ARNTL complex. Another regulatory loop is induced by the CLOCK:ARNTL complex activating the transcription of *nr1d1* and *rorc* genes (e.g., *rorca* and *rorcb*), which in turn both positively and negatively regulate the transcription of *arntl* (see Fig 2B). Many of the genes that transcribe these activators and repressors show reductions or loss of cycling in one or more cave populations, and a few show reductions in rhythmicity in all three caves (e.g., *rorca*, *rorcb*, *arntl2*)(Table 1 and Fig 2). While reductions in rhythmicity at core clock genes support an overall dampening of the core circadian mechanism in cave populations compared to surface forms, several core clock genes show a dampening or loss of rhythmic expression in only one cave population (Fig 2).

**Table 1:** Known circadian regulators with losses or reductions in rhythmicity in cave populations^1^.

Gene name	Disrupted Populations	Predicted functions of transcribed proteins
***Core loop***		
*arntl1a*	Tinaja, Pachón	Activator in core circadian feedback loop[54]
*arntl1b*	Tinaja, Pachón	Activator in core circadian feedback loop[54]
*arntl2*	Tinaja, Molino, Pachón	Activator in core circadian feedback loop[54]
*cry1aa*	Pachón	Repressor in core circadian feedback loop[55]
*cry1ba*	Tinaja, Pachón	Repressor in core circadian feedback loop[55]
*cry1bb*	Tinaja, Pachón	Repressor in core circadian feedback loop[55]
*cry4*	Pachón	Potential photoreceptor function[9,55]
***Accessory pathways***		
*rorca*	Molino, Tinaja, Pachón	Regulator of core feedback loop[56]
*rorcb*	Molino, Tinaja, Pachón	Regulator of core feedback loop[56]
*bhlhe40*	Molino	Regulator of core feedback loop[57]
*bhlhe41*	Tinaja, Pachón	Regulator of core feedback loop[57]
*fbxl3a*	Molino, Tinaja, Pachón	Regulator of core feedback loop[58,59]
*nfil3*	Tinaja, Pachón	Regulator of core feedback loop[60]
*nfil3-5*	Tinaja, Pachón	Regulator of core feedback loop[52]
*cipcb*	Pachón	Regulator of core feedback loop[61]
***Mediators of downstream physiology and behavior***		
*aanat2*	Molino, Tinaja, Pachón	Controls daily changes in melatonin production[62]
*camk1gb*	Molino, Tinaja, Pachón	Involved in linking pineal master clock with peripheral circadian activity[63]
*nptx2b*	Pachón, Molino	Modulates circadian synaptic changes[64]

^1^Exhaustive list of genes with changes in rhythmicity can be found in S1 Data.

Genes with reduced rhythmicity in cavefish populations also include those implicated in the regulation of oscillations in physiological and behavioral rhythms (Table 1) and non-visual or extra-ocular opsins (e.g., *exorh*, *parapinopsinb*, *opn6b*). Among the genes with reductions in rhythmicity across all three cave populations is *arylalkylamine N-acetyltransferase 2* (*aanat2*), a key regulator of melatonin synthesis in the pineal gland [62,67,68]. Zebrafish *aanat2* mutants sleep less at night, suggesting a critical role in circadian regulation of sleep [62]. Like in zebrafish [62,67], the expression of *aanat2* in surface *A*. *mexicanus* shows robust rhythmic behavior (*q* = 6.6 x 10^−4^) with peak expression during subjective night. In contrast, cavefish do not show evidence of rhythmic transcription of *aanat2* (Molino, *p =* 1.0; Tinaja, *p* = 0.82; Pachón, *p* = 0.33) and expression does not increase in these populations during subjective night (Fig 2). Another gene that shows reductions in rhythmicity across all three caves is *camk1gb*, which is involved in linking the pineal master clock with downstream physiology of the pineal gland. Knock-downs of this gene in zebrafish significantly disrupt circadian activity[63], similar to what is observed in Pachón cavefish [45,46]. *Camk1gb* also plays a role in regulating the rhythmic transcription of *aanat2*; knockdowns of *camk1gb* reduce the amplitude of *aanat2* expression rhythm by half in zebrafish but do not affect transcription of the core clock [63]. These findings suggest changes in transcriptional regulation of genes downstream of the core-clock potentially impact loss of rhythmic behaviors in cavefish.

### Rhythmic transcripts in cave populations show a delay in phase compared to the surface population

The timing of transcriptional oscillations is key to matching circadian rhythms and environmental cycles. To determine whether the pace of cycling is changed across *A*. *mexicanus* populations, we investigated whether the timing of the circadian clock differs between cave and surface populations by comparing the phase of cycling transcripts between populations.

**Fig 3:**
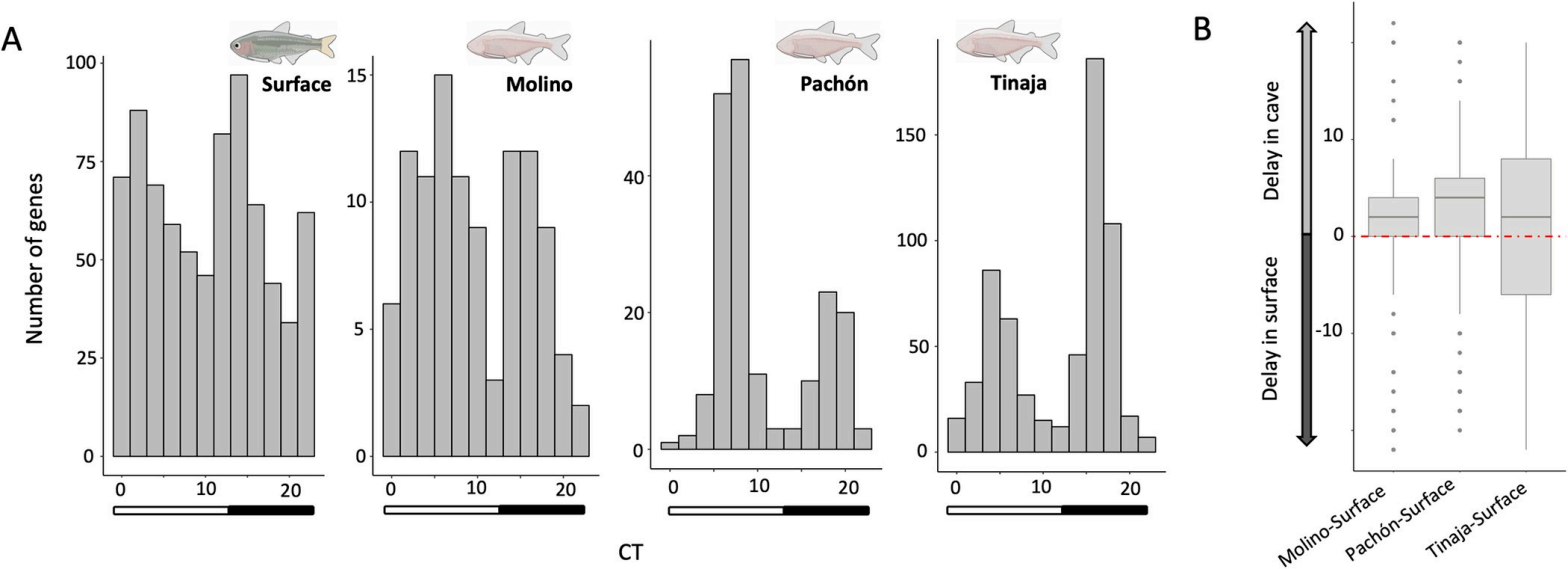
A. Distribution of peak expression time of rhythmic transcripts in each population. Bars indicate subjective day and night. B. Cycling genes on average show a delay in phase in cave populations relative to their phase in the surface population.

Consistent with the bimodal distribution of circadian phases seen in other systems [52,69], most transcripts in surface and cave populations of *A*. *mexicanus* show peak expression before subjective dawn or dusk (see Fig 3A). However, this distribution was shifted towards later in the subjective day in fish from all three cave populations compared to the surface population. This transcriptome-wide shift is consistent with the individual gene analysis of Beale *et al*. [24], which showed that the clock genes *per1* and *cry1a* display phase delays in fish from the Pachón and Chica caves relative to surface fish (Fig F in S1 Text)(see also [70]).

We then compared the phase of *A*. *mexicanus* genes with phase estimates of orthologous core clock genes in zebrafish sampled under dark conditions [52]. While zebrafish and *A*. *mexicanus* diverged 146 MYA [71], the timing of peak expression of core clock genes was highly similar in zebrafish and surface fish (median difference of 0.8 hours between orthologs, Table D in S1 Text). Comparatively, the phase of clock genes in the core and accessory loops are often more shifted in cave populations relative to zebrafish phase than the surface population (median shifts of 4.7, 2.2, and 3.5 hours for Tinaja, Molino, and Pachón, respectively)(Table D in S1 Text). Thus, the phase of core clock and accessory loops appears well-conserved over a long evolutionary timespan between zebrafish and surface *Astyanax*, yet appears to be dramatically shifted in cave populations.

To further characterize differences in phase between surface and cave populations, we compared the phase of all genes, including genes in core and accessory loops from above, with evidence for rhythmic expression across cave-surface population pairs (where *p* < 0.05 for a gene in the surface and the cave population being compared). Consistent with the phase shifts observed for genes in the core clock and accessory loops, for each cave-surface comparison, we found that rhythmic transcripts showed significantly later peak expression in cave populations (Wilcoxon signed rank test, all pairwise comparisons *p <* 2.2 x 10^−16^; average shift, surface-Pachón: 2.03 hours, surface-Tinaja: 1.3 hours, surface-Molino: 0.48 hours; Table E in S1 Text)(Fig 3B, phase of all genes in S1 Data).

### Genes with circadian *cis*- elements show loss of cycling despite maintenance of motifs in cave populations

Since *cis-*regulatory elements often drive expression differences, we investigated whether these critical regulators exhibit disruptions as in the circadian transcriptome. In vertebrates, circadian oscillations in gene expression are a consequence of interlocking transcriptional feedback loops of core clock proteins binding to a combination of known *cis-*elements (E-box, RRE, D-box) in the promoters and enhancers of target genes [52,72]. E- and D-box elements also play a critical role in light-dependent clock activation [73,74]. The timing of activator and repressor binding to circadian *cis-* elements results in the phase of oscillating transcripts [72].

To understand if the likely functions of key circadian regulatory motifs are conserved between surface *A*. *mexicanus* and zebrafish, we extracted promoter sequences of all genes with rhythmic transcription in the surface population and identified transcription factor (TF) binding motifs in each sequence (Table F in S1 Text). We consider rhythmic genes with significant circadian binding motifs (i.e., E-box, RRE, D-box sequences) putative targets of clock proteins in the circadian feedback loop. We then used a sliding window approach to identify the specific time window when the phases of circadian TF targets are most enriched in surface fish. Consistent with observations in zebrafish [52], E-box motifs, which are bound by the CLOCK-ARNTL complexes as part of the core feedback loop [75], were enriched in the promoters of genes with peak expression in the morning (CT 2), where RRE elements, which are bound by RORA proteins and NR1D1/NR1D2 of the accessory loop[76], were enriched in the promoters of genes with peak expression in the evening (CT 14–16)(Fig G in S1 Text)(see Methods). The D-box, which binds bZip factors including NFIL3, was also enriched in promoters of genes with peak expression in the morning (CT 0–6), as in zebrafish [52] (Fig H in S1 Text). Thus, our analysis suggests that key circadian regulatory motifs, and subsequently, the timing of key regulatory cascades mediating circadian oscillations in gene expression, are largely conserved between *A*. *mexicanus* surface fish and zebrafish.

Next, to understand if divergence in *cis-* regulatory elements may contribute to dysregulation of circadian gene expression in cavefish, we compared cave and surface fish promoter sequences. First, we searched for circadian *cis-* elements in cavefish sequences upstream of genes that have lost rhythmicity in cave populations. A large proportion of the putative targets of core clock elements identified in surface fish (e.g., rhythmic genes identified as having an E-box, RRE or D-box motifs in their promoter sequence, as described above) do not cycle in one or more cave populations: >77% are arrhythmic in at least one cave population. We tested whether the loss of circadian transcription can be explained by a reduction in circadian motifs, however, we found no evidence for an enrichment of binding motifs in the promoter sequences of these ancestrally rhythmic genes in the surface fish compared to cave populations (*p* = 1 for all tested motifs, see S1 Text). Further, for nearly all genes for which we identified a proximal circadian motif in the surface fish, we also identified a binding site in cavefish (but see Table G S1 Text for cases of motif loss). In sum, our analysis found that was (1) there were no differences in the enrichment of binding sites between cave and surface promoter sequences in ancestrally rhythmic genes, and (2) there were few cases where putative *cis-* regulatory sequences identified in surface fish were disrupted in cave populations. Consequently, our data support that the reduction in the number of rhythmic transcripts in cavefish may be a consequence of changes in the transcription of core clock genes or light-input pathway rather than divergence at the target *cis-* binding sites of downstream genes (e.g., *trans-* effects or *cis*-by-*trans* effects).

### Genetic differentiation at clock genes between cave and surface populations

Having characterized widespread transcription dysregulation of the circadian transcriptome in cavefish, we searched for evidence of exceptional allele frequency differences between cave and surface populations among circadian genes. Population genomic metrics were calculated for the three cave populations, Molino (n = 9), Pachón (n = 10), Tinaja (n = 10), the Río Choy surface population (n = 9), and one additional surface population (Rascón, n = 8) for which population genomic data is available. We found that of 416 genes which showed reduced rhythmicity in expression in at least one cave population, 77 were in the largest 5% of *F*_*ST*_ values across all genes in the genome for at least one cave-surface comparison. These outliers included genes with known roles in circadian regulation (e.g., *cry1ba*, *exorh*) (S1 Data).

If circadian rhythms are dispensable in the cave environment, we may observe a relaxation of purifying selection in the protein-coding sequences of clock genes. In contrast, clock genes can also be important regulators of other processes (e.g., [77,78]), and so coding sequences may remain under strong purifying selection as a consequence of pleiotropy. To address the role of relaxed selection in clock evolution, we aligned cave and surface *A*. *mexicanus* sequences from 18 genes from the core and accessory loop, as well as *aanat2* and *exorh*, with one-to-one orthologs of up to 23 teleost species (see S1 Data and Fig W in S1 Text). Aligned coding sequences were used to test for changes in selection intensity in lineages leading to cavefish populations (Molino and Pachón/Tinaja) under a phylogenetic framework by comparing ω (ratio of nonsynonymous (dN) to synonymous (dS) nucleotide substitution) and the selection strength parameter *k* through branch-site random effects models [79] (S1 Text). In the core clock, we found *per1b* (*p* = 0.0004), *cry4* (*p =* 0.0002), *arntl2* (*p =* 0.025), and *cry1ab* (*p* = 0.048) show evidence of relaxed constraint in Tinaja and Pachón. *Exorh*, the non-visual opsin, was also found to be under relaxed constraint in Tinaja and Pachón (*p* = 0.00015). We found no evidence for relaxed constraint in clock sequences of Molino. Comparing this to 1,000 random permutations of 18 randomly selected orthologs, we found that the number of clock genes with evidence for relaxed constraint in Pachón and Molino is more than expected by random sampling (*p* = 0.021).

Within circadian regulators with evidence for relaxed selection, we identified variants computationally predicted to be deleterious to protein function. We identified variants at high frequency (> = 0.8) in one or more cave populations but absent in surface populations, and then used *in silico* prediction algorithms SIFT and VEP to assess their potential impact on protein function (see S2 Data and S3 Data for full list of predictions). *Cry4* were found to harbor a deleterious nonsynonymous substitution (Chr22:17502170 N>Y) and a predicted frameshift (Chr22:17504874–17504875) in Pachón and Tinaja. *Per1b* was found to contain two high frequency deleterious variants in Tinaja (Chr16:34045043 R>W; Chr16:34055448 P>R). Finally, *exorh* contained predicted deleterious substitutions in Tinaja and Pachón (Pachón: Chr6:45608103 L>I, Chr6:45608179 A>V; Tinaja: Chr6:45608103 L>I).

### Visualization of key circadian mRNAs reveals tissue-specific expression patterns in cave and surface populations

Our RNAseq data suggests that rhythms in the core clock are dysregulated, and that rhythmic transcripts are phase-shifted in cavefish populations. Teleost circadian systems are highly decentralized and autonomous core clock gene expression may be observed in many tissues. Consequently, we tested whether circadian dysregulation manifests differently in different tissue types in cave populations. To characterize temporal expression of clock genes across different tissues in cave and surface forms, we used RNAscope fluorescence in situ hybridization (FISH)[80]. We collected brains and livers from individual 30dpf fry at three timepoints (CT0, CT8, and CT16)[81] and performed RNA FISH for key circadian mRNAs in the primary loop (*per1* and *arntl1a*) and regulatory loop (*rorca* and *rorcb*)(see Methods for details and controls)(Figs I-Q in S1 Text).

**Fig 4:**
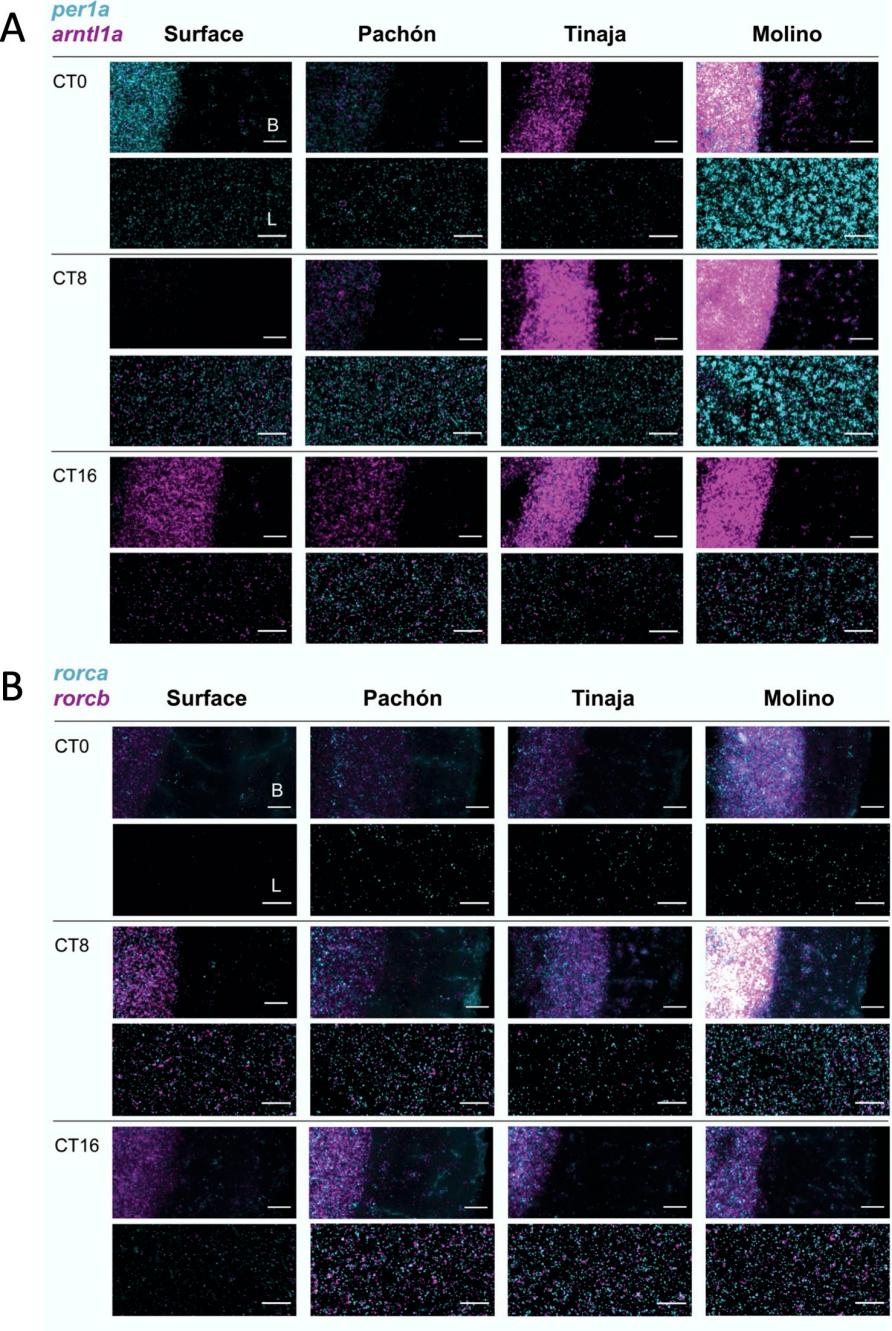
Temporal expression patterns of (A) *per1a* (cyan) and *arntl1a* (magenta) and (B) *rorca* (cyan) and *rorcb* (magenta) in brain and liver tissue in *Astyanax mexicanus* populations. In-situ staining using RNAscope in the midbrain (‘B’, top panels for each timepoint) and liver (‘L’, bottom panels for each timepoint) of surface fish and cavefish (Pachón, Tinaja, Molino) at CT0, CT8, and CT16. Each time point is a single fish sample with probes separated into two channels. Images are representative sections of two fish collected per time point, per population. Scale bar is 25μM.

**Fig 5:**
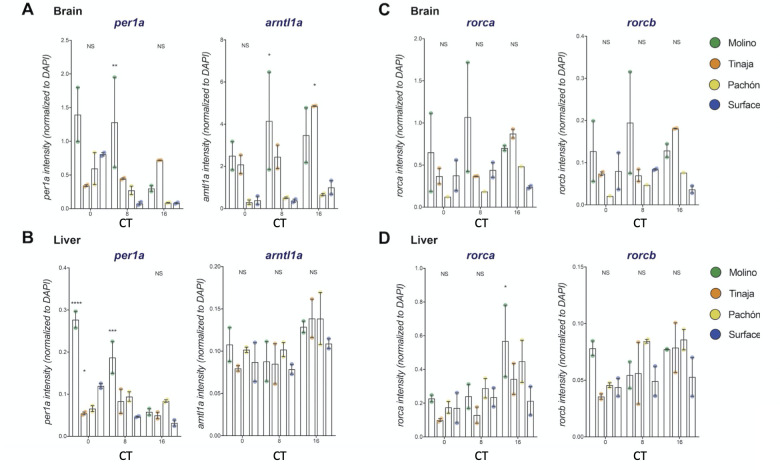
Quantification of RNA FISH. A-D. Expression of *per1a*, *arntl1a*, *rorca* and *rorcb* in brains (A, C) and livers (B, D) at CT0, CT8, and CT16 in surface fish and cavefish populations. RNAscope probe channel intensity was normalized to DAPI channel intensity in identically sized, anatomically matched ROIs to provide an estimate of mRNA expression per cell (see Methods for full details of RNA FISH analysis). Biological replicates are shown as colored points on graph and represent a brain or liver sample collected from a single individual. Bars reflect mean and error bars show SEM of biological replicates. Statistics were calculated for each mRNA probe by comparing each cave population mean to control mean (Surface) within timepoints using ordinary 2-way ANOVA. Dunnett’s test was used to correct for multiple comparisons across populations, timepoints. Adjusted p-values < 0.05 are reported with * using the following scheme: 0.0332 (*), 0.0021 (**), 0.0002 (***), and <0.0001 (****).

We find that temporal expression patterns of *per1a* and *arntl1a* mRNA in the midbrains of surface fish are consistent with our RNAseq results and expression patterns in zebrafish[52] (Fig 4B and Figs N and O in S1 Text). *Per1a* expression peaks strongly at dawn (CT0) and *arntl1a* cycles in anti-phase and shows high expression at dusk (CT16). Also consistent with our observations in whole fry, we see a number of population-specific alterations to the expression of these genes in cave populations. In cave populations, *per1a* shows broader temporal expression and persists later into the day (Figs F and N in S1 Text). *Arntl1a* was also found to be expressed more broadly in Tinaja and Molino outside of CT16, the primary window of expression in surface populations. The Molino midbrain also showed high basal levels of expression compared to the surface, particularly at CT8, when *per1a* and *arntla* expression is nearly absent in surface fish (Fig 5 and Fig L in S1 Text).

Temporal expression patterns for *per1a* and *arntl1a* in surface fish liver are similar to that of the brain. In contrast, peak and trough expression in Pachón and Tinaja livers appear to be out of sync with those in the midbrains (Fig 4A, ‘L’ vs. ‘B’). *Per1a* expression in Tinaja liver is greatest at CT8, but is expressed highest in brains at CT16. In Pachón midbrains, *per1a* expression is highest at CT0, but expression is highest in the livers at CT8 and CT16.

Next, we examined the timing of *rorca* and *rorcb* expression, two members of the regulatory loop that regulate the expression of *arntl* paralogs and circadian transcription [76]. Our RNAseq results indicate that *rorca* and *rorcb* show peak expression midday and minimal expression at CT0 or CT16 in whole fry in surface fish (Fig 2), similar to what has been observed in zebrafish (Table D in S1 Text). Consistent with this, RNA FISH of surface fish midbrains showed that *rorca* and *rorcb* expression was greater midday on average between replicates (Fig 5C and Figs M, P, and Q in S1 Text). When compared to surface tissues, the expression of *rorca* and *rorcb* in Tinaja and Pachón brains and livers peaked later (CT16). Molino, by contrast, showed higher expression in the evening for liver and midday for the brain for *rorca* and *rorcb*, though expression was highly variable between replicates (Fig 5C vs. 5D). Again, Molino also showed high basal expression in the midbrain relative to other populations.

Thus, as observed in the whole organism RNAseq analysis, tissue-specific expression of these primary loop and regulatory loop genes confirm the phase shifts and dysregulation of cavefish transcripts compared to surface fish, though our RNA FISH analysis suggests that disruptions to the temporal regulation of *per1a* and *arntl1a* expression manifests differently across tissues in Pachón, Tinaja, and Molino cavefish (Fig 4).

### Melatonin rhythms differ between cave and surface fish

Melatonin is a key hormone and behavioral regulator in the circadian system of vertebrates. In most fish, daily melatonin fluctuations are regulated in part by *aanat2*: increased *aanat2* mRNA expression levels during the second half of the day results in high AANAT2 protein levels at night and increased synthesis of melatonin [62,82]. As *aanat2* did not show rhythmic transcription or an increase during the subjective night in cavefish populations (Fig 2), we tested whether melatonin rhythms differ between cave and surface populations.

**Fig 6:**
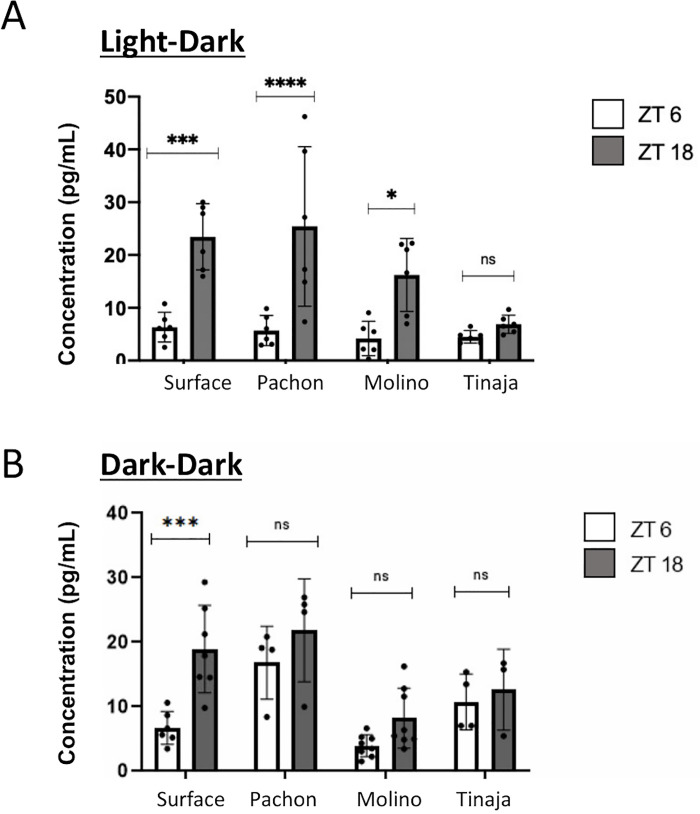
A. Melatonin under light-dark conditions. Ten fish larvae were pooled together, homogenized and melatonin was extracted for each datapoint. Melatonin increased at night in Surface fish, Pachón and Molino population (two-way ANOVA analysis). There was no significant change in Tinaja cavefish. *, *p* = 0.0117; *** *p* = 0.0002; **** *p*<0.0001. B. Melatonin under dark-dark conditions. Ten fish larvae were pooled together, homogenized and melatonin was extracted for each data point. Melatonin increased at night in Surface fish population (two-way ANOVA analysis). There was no significant change in Tinaja, Pachón, or Molino cavefish. ***p-value = 0.0004.

To determine whether melatonin rhythms differ between cave and surface fish, melatonin levels were measured in surface and cave fish under light-dark and dark-dark conditions in the morning (ZT 6) and evening (ZT 18). Under light-dark conditions, melatonin increased at night in surface fish (two-way ANOVA, *p* = 0.0002), Pachón (*p* < 0.0001), and Molino (*p* = 0.012) individuals. We found no significant difference between evening and morning levels of melatonin for Tinaja (Fig 6A). In contrast, under dark-dark conditions, melatonin increased at night in surface fish (*p* = 0.0004), but not in any of the cavefish populations (Fig 6B), consistent the lack of rhythmicity of *aanat2* mRNA expression in these populations. Consequently, our results suggest that while Pachón and Molino cavefish can produce melatonin rhythms under light-dark conditions, endogenous melatonin rhythms are lost under constant darkness. Tinaja cavefish, by contrast, appear to have lost melatonin rhythms altogether, suggesting functional differences in the loss of circadian function across cavefish populations.

### *aanat2* and *rorca* regulate sleep behavior in surface fish

Circadian rhythms influence a variety of physiological and behavioral traits, including regulation of the sleep-wake cycle [81]. Our analyses indicate the core clock genes and the melatonin regulator *aanat2* are dysregulated on the transcriptional level, and that melatonin cycling is disrupted under dark conditions in cave populations. *Aanat2* transcription and melatonin synthesis and are required for the circadian regulation of sleep [62], raising the possibility that evolved differences in circadian transcription underlies the loss of sleep in cavefish [34]. Consequently, we sought to test the role of *aanat2* in the timing of locomotor activity and sleep.

**Fig 7:**
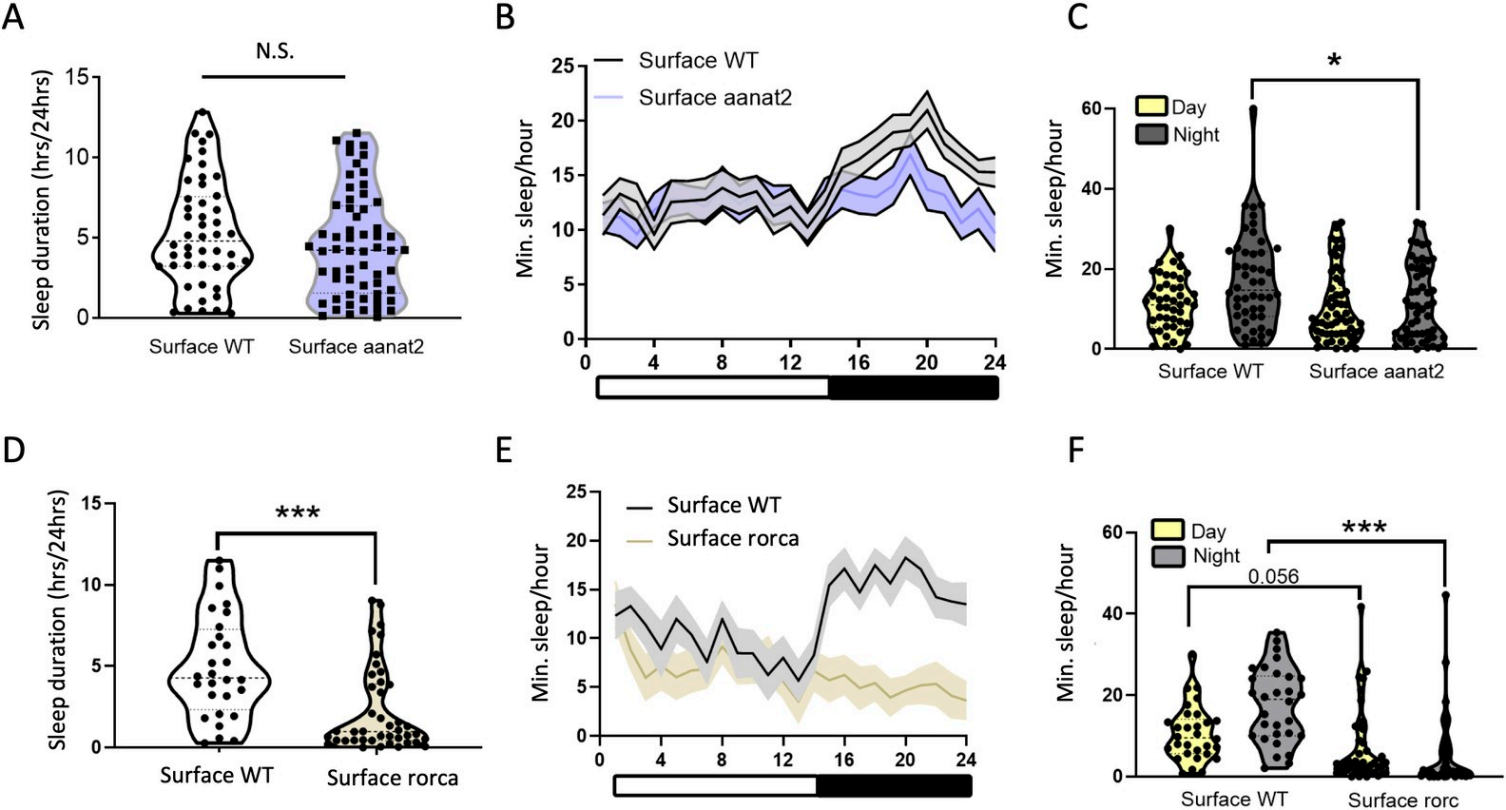
Mutant *aanat2* and *rorca* fish reveal a role for these genes in sleep behavior in *A*. *mexicanus*. A. Total sleep is not significantly altered between control and *aanat2* crispant surface fish (Mann-Whitney U, *p* = 0.99). B-C. Day sleep is not significantly altered between WT and crispant *aanat2* fish (Mann-Whitney U, *p* = 0.22). Night sleep is significantly reduced in *aanat2* crispants compared to WT controls (Mann-Whitney U, *p* = 0.015). D. Total sleep is significantly reduced in crispant *rorca* fish compared to WT controls (Mann-Whitney U, *p*<0.0001). E-F. Day sleep was not significantly reduced between WT and *rorca* crispants (unpaired t-test, *p* = 0.56). Night sleep is significantly reduced in *rorca* crispants compared to WT controls (Mann-Whitney U, *p*<0.0001).

To understand the role of *aanat2* in rest-activity regulation in *A*. *mexicanus*, we generated surface fish mosaic for mutations in *aanat2* (crispants) using CRISPR/Cas9 (see Methods). Crispant 30 dpf fish and wildtype (WT) sibling controls were phenotyped for sleep and locomotor activity as previously described [44,83]. Night sleep duration (minutes/hour) was significantly reduced in *aanat2* crispants (Mann-Whitney U, *p* = 0.015) but crispants and WT fish slept the same amount per 24-hr period (Fig 7B and 7C). In light of reduced RNAseq expression of *aanat2* during the subjective night in all cavefish populations, our crispant results suggests differences in *aanat2* expression may contribute to reduced nighttime sleep observed in cavefish.

Transcription of the core clock genes were also disrupted in cavefish populations, with some core clock genes (e.g., *rorca*) showing reductions or losses of rhythmic expression in all three cave populations. To examine the functional consequences of the repeated evolution of the disruption of one such genes, we created crispants for *rorca*. *Rorca* crispants were found to sleep significantly less minutes per hour at night compared to WT sibling fish (Mann-Whitney U, *p* < 0.0001, Fig 7E and 7F). Further, *rorca* crispants also sleep less overall during a 24-hr period (Mann-Whitney U, *p* < 0.0001, Fig 7D). While WT surface fish sleep an average of 5.16 hours per 24-hr period, *rorca* surface crispants sleep just 2.26 hours per 24-hr period. Consequently, the repeatedly evolved transcriptional dysregulation seen in the core clock in cavefish populations may also contribute to the evolution of sleep loss observed in cavefish populations.

## Discussion

Circadian rhythms are nearly ubiquitous in eukaryotes, directing aspects of behavior, physiology, and metabolism [2]. The biological clock is thought to provide a mechanism for organisms to synchronize their physiology and behavior to predictable daily cycles [3,4]. However, we have a limited understanding of how the biological clock is altered in the face of arrhythmic environments, where organisms are isolated from regular environmental cues, and how this impacts downstream physiology and behavior [8,21,24,84,85]. Here, we demonstrate that replicate, independent origins of the cave phenotype of *A*. *mexicanu*s allow for an unprecedented opportunity to examine how clocks are dysregulated in arrhythmic environments. We identified many repeated features of circadian dysregulation across populations (e.g., loss of oscillations of transcripts in the core clock, shifts in circadian phase) and also highly population-specific circadian clock dysregulation.

Clock dysregulation and relaxed selection on clock genes supported the largely independent loss of the molecular basis for circadian rhythms among different caves. For example, Molino and Pachón cave populations exhibited less than a 1/3^rd^ of cycling transcripts in Tinaja cavefish. Our RNA FISH analysis also indicated population-specific decoupling of rhythms for genes in the core loop (*arntl1a*, *per1a*) in cave populations compared to surface fish in different tissues. In fish, most tissues and cells have independent, light-responsive pacemakers [14,86], and previous work on *A*. *mexicanus* sampled fins clips [24]. Tissue-specificity can contribute to dysregulation patterns of specific circadian genes surveyed in our RNAseq dataset. While these population-specific features may be stochastic, clock evolution in different caves may also be influenced by cave-specific non-visual zeitgebers, such as the presence of food or other rhythmic animals [22]. Currently, no evidence supports bat activity, specifically, as a zeitgebers for cavefish [24], though future work focused on environmental cues within specific caves could illuminate what environmental factors impact circadian clock evolution in this system.

Changes in the phase of circadian genes between surface and cavefish populations suggests differences in entrainment between populations. One potential mechanism for phase delay is divergence in the light-input pathway, for which circadian non-visual and extra-ocular opsins are potential candidates [70]. Rhythmic expression was lost or significantly reduced for several opsins, including extra-ocular opsin *exo-rhodopsin* (*exorh*). In zebrafish, this gene plays important role in mediating the effects of environmental light on pineal rhythms and melatonin synthesis through the regulation of *aanat2*. *Exorh* depletion results in a significant reduction of *aanat2* transcription, suggesting it may be responsible for initiating *aanat2* transcription in response to lighting conditions [87]. *Exorh* sequences were highly differentiated, with evidence for relaxed selection, between the Pachón and Tinaja cave populations and surface populations. Non-visual opsin mutants have been found to play an important role in mediating the peripheral clock light input pathway of the Somalian blind cavefish [22], making mutations in non-visual opsins interesting candidates for changes in clock regulation in *Astyanax* cavefish.

An important open question is how dysregulation of the circadian transcriptome has affected downstream phenotypes in cavefish and what consequences these changes have for organismal fitness. Cave and surface fish populations show differences in locomotor and metabolic rhythms under constant darkness [24,43]. We hypothesize that circadian clock dysregulation also plays a role in the evolved sleep loss in cave populations [34]. While the biological clock plays an important role in regulating the sleep-wake cycle, clock evolution has never been associated with evolved sleep differences between populations or species. In addition to repeated alterations across cave populations in the core and accessory loops (e.g., *rorca*, *rorcb*, *arntl2*), we also found that *aanat2*, a regulator of melatonin synthesis in the pineal gland, showed reductions in rhythmicity across all three cave populations. Cavefish did not show increased *aanat2* expression during the subjective night (Fig 2), in contrast to surface fish and other teleosts [82]. The rise of *aanat2* mRNA levels at night is associated with increased melatonin levels, and the onset of nighttime sleep [62]. Further, melatonin levels were found to be the same in cavefish in the morning and evening in dark-dark conditions (Fig 6). The diminished nighttime sleep we observed in *aanat2* crispants demonstrates that when *aanat2* is not transcribed, nighttime sleep duration is significantly reduced in *A*. *mexicanus*. This result demonstrates that evolved alterations to circadian rhythms in expression of genes that output from the core clock could impact evolution of sleep. Our data showed dampened *rorca* rhythmic expression in all cave populations, and crispants indicated *rorca* plays a role in regulating sleep length overall. Consequently, we hypothesize that the dysregulation of the core clock and downstream circadian regulators of the sleep-wake cycle contribute to the repeated evolution of reduced sleep duration in cavefish.

The loss of environmental light has implications for organismal fitness beyond circadian physiology. Environmental light plays an essential role in cell cycle control and activating DNA repair in teleosts. Previous work in this system found that cave populations of *A*. *mexicanus* show higher levels of DNA repair activity and increased expression of DNA repair genes in the dark [24]. As a common light signaling pathway controls both DNA repair and clock entrainment, one proposed mechanism is that cavefish have sustained upregulation of clock genes normally driven by light, akin to ‘perceiving’ sustained light exposure [24]. We found that DNA repair genes are more often upregulated in whole fry from cave populations (Table J in S1 Text), and genes involved in DNA repair showed enhanced or uniquely rhythmic expression in Molino and Tinaja (see S1 Text). However, genes associated with light induction were not more likely to be upregulated in cavefish compared to surface fish under dark-dark conditions (Table K in S1 Text). Light-activated genes in the core circadian feedback loop were also not consistently upregulated in cave populations (see S1 Text). Thus, while our analyses support changes to the DNA repair system in cavefish, specifically in Molino and Tinaja, more work is necessary to understand the relationship between the light input pathway, clock evolution, and the regulation of DNA repair in this system.

Against the backdrop of independent dysregulations of the circadian transcriptome, we identified a common set of genes with significant rhythmic expression across all cave and surface populations. These include genes in the primary (e.g., *per1a*, *per1b*) and accessory loops (e.g., *dbpb*, *cipca*). These genes may represent a subset of clock components that are minimally required to maintain some endogenous rhythmic physiology, or may be under strong constraint for their role in other organismal functions. Alternatively, the shared sustained rhythms of these genes may be a shared stochastic feature of a dysregulated circadian clock. *Per1b*, for example, despite its sustained rhythmic activity, shows evidence of relaxed selection and harbors predicted deleterious substitutions.

Altogether, this study has provided novel and significant insights at the intersection of evolutionary biology and chronobiology. We have demonstrated that in the face of the loss of key environmental zeitgebers, three cave populations have evolved widespread disruptions to the circadian transcriptome. Our analyses support a model where disruptions to the biological clock have evolved independently and by different molecular mechanisms in different populations. Consequently, our results demonstrate that movement into largely arrhythmic environments, like caves, can result in predictable dampening or losses in endogenous transcriptional rhythms, even on relatively short evolutionary timescales, and such losses of circadian function may have impacts on sleep phenotypes.

## Methods

### Ethics statement

Experiments were approved by the Institutional Animal Care and Use Committee at Florida Atlantic University (Protocols #A15-32 and #A18-38) and the Institutional Animal Care and Use Committee (IACUC) of the Stowers Institute for Medical Research (institutional authorization 2019–084).

### Sampling

To understand the extent to which cave populations maintain a functional biological clock and compare cave and surface fish transcriptomes, samples from each population were derived from the same lab-born mating clutch and raised under a 14:10 light-dark cycle. Light-dark conditions mimic the ancestral condition of cavefish and raising all fish under these conditions allows us to infer evolved differences in the circadian transcriptome between cave and surface populations[70]. Experimental individuals were descended from non-inbred lab-born individuals. To avoid food entrainment, enough food was added to tanks so that food was always available. Additional food was added each day in a 2-3hr window. All fish were exactly 30 days post fertilization at the start of the experiment. Fish were kept in total darkness for 24-hours prior to sampling and throughout the duration of the experiment. During the experiment, food was added twice daily, in the morning and evening (exactly at 8am and 8pm), however, food was available at all times because most was not immediately consumed. Six replicates were first sampled at 6am (Circadian Time 0) and then every four hours until 2am (corresponding to CT 20). Individuals were immediately flash frozen in liquid nitrogen. RNA was extracted from the whole organism with extraction batch randomized for time of sampling and population. Samples were sequenced on the Illumina HiSeq 2500 platform to produce 125-bp paired-end reads (S1 Data) using strand-specific library preparations. Samples were randomized across sequencing lanes relative to population and time of sampling. Previous work showed no extraction batch or lane effect on these samples [88].

### Read mapping

Reads were cleaned of adapter contamination and low-quality bases with Trimmomatic (v0.33)[89]. Cleaned reads were mapped to the *A*. *mexicanus* draft genome assembly v1.02 (GCA_000372685.1) with STAR [90] and reads overlapping exonic regions were counted with Stringtie (v1.3.3d)[91] based on the *A*. *mexicanus* Ensembl v91 annotation. Genes with fewer than 100 reads across all samples were removed from the analysis. Reads were subsequently normalized for library size and transformed with a variance stabilizing transformation with DESeq2[92] for principle component analysis.

### Identification of rhythmic transcripts

Rhythmic transcripts with 24-h periodicity were identified using the program JTK_cycle[51], using one 24-h cycle, with a spacing of 4 hours and 6 replicates per time point. JTK_cycle was also used to estimate the amplitude and phase of each rhythmic transcript. We retained genes as rhythmic at a False Discovery Rate (FDR) of 5%. Our overall result, that more genes are cycling in the surface population than in cave populations (Table A in S1 Text), was insensitive to specific value of the FDR cut-off. We defined arrhythmic transcripts at *p*-value > 0.5, a conservative cut-off for arrhythmic expression that has been used in other studies [66,93].

An important caveat to our analysis of rhythmic expression is that cave and surface forms differ in their proportion of retina tissue on 30 dpf. However, due to the decentralized nature of the teleost biological clock, where tissues have independent, light-responsive pacemakers, this will predominately affect transcripts that are rhythmic only or predominantly in the retina. As most circadian genes cycle in multiple tissues in fish (including the primary and accessory feedback loops), our comparison will be able to address evolved differences in rhythmicity in these transcripts. Consistent with this notion, much of the dysregulation we have found in circadian transcription of cave populations is population-specific. Population-specific losses of rhythmic expression are not expected to be predominant if dysregulation is due to loss of retinal tissue alone.

### Differential rhythmicity analysis

To identify differential rhythmicity between pairs of populations, we calculated a differential rhythmicity score (S_DR_) for genes with a JTK_cycle *p*-value < 1 in either population. As in Kuintzle *et al*.[66], S_DR_ was defined as:

$$S_{DR}=\frac{Z_{P}+Z_{R}}{\sqrt{2}}$$

Where Z_p_ is the Z-score for changes in periodicity between populations, where changes in periodicity are defined as log(*p* -value of population 1)–log(*p* -value of population 2). Z_R_ is the Z-score computed for changes in amplitude between populations, where changes in amplitude are defined as: log_2_(Amplitude of population 2 / Amplitude of population 1). Amplitude for each gene was estimated with JTK_cycle.

We then computed a *p*-value for S_DR_ values using a Gaussian distribution based on the fit to the empirical distribution. We used R’s *p*.*adjust* to perform a Benjamini & Hochberg correction for multiple testing (Table I in S1 Text).

### Promoter analysis of rhythmic genes

We defined putative promoters as the region 1-kb upstream to 200bp downstream from the transcription start site (TSS)[52]. Population genetic data[50] was used to identify variants with putative promoter regions segregating between populations and create alternative reference genomes for each population. Genes with a JTK_cycle FDR < 0.1 in the surface were used to identify phase enrichment of circadian *cis-* elements in the surface population. FIMO of the MEME suite[94] was used to identify motifs in each promoter. FIMO was used to estimate *p*-values for motifs in each sequence; motifs with *p*-values < 0.0001 (the default cut-off) were considered for downstream analyses. Notably, more distal *cis-* regulatory elements will be excluded from this scan. To identify phase-specific enrichment of binding motifs, we tested sliding windows of time with Fisher’s exact tests for motifs in phase versus motifs out of phase compared to the total set of in- and out- of phase genes. To compute changes in the phase of genes that are putative targets of circadian transcription factors between surface and cave populations, we calculated the minimum distance between the peak expression between cave and surface populations based on a 24-h clock. Further details of this analysis are available in the S1 Text.

### Tissue preparation for RNA fluorescence in situ hybridization (FISH)

Pachón, Molino, Tinaja, and surface fish derived from the Río Choy population were raised in densities of 20–25 individuals/3L tank in 14:10LD cycle at 23°C and 750–800μS/cm conductivity, with feeding and water quality control as previously described[95].

To make RNA FISH comparable to our RNAseq analysis, imaging was performed on fry 30dpf. 29 dpf fish were placed in constant darkness in the dark phase of the day preceding tissue collection. 30dpf fish were fed *ad libitum*, with additional food added at 8am and 8pm on the day of tissue collection. Individual fish were euthanized in MS-222 at CT0, CT8, and CT16. Experiments were performed in duplicate: two fish were euthanized and dissected at each time point from each population. All efforts were made to reduce light exposure prior to fixation. Dissected brains and livers were placed into a freshly prepared solution of 4% PFA in DEPC 1X PBS on ice. Samples were transferred to room temperature after 3–5 minutes on ice and allowed to fix for 1 hour. Samples were then placed at 4°C for 36 hours. After 36 hours, samples were rinsed briefly in DEPC water and washed in DEPC 1X PBS three times for 15 minutes with constant shaking. A graded EtOH wash was performed (30%- 50%- 70%- 100%) in DEPC treated water with 5 minutes between steps.

Tissue processing and paraffin embedding were performed with a PATHOS Delta hybrid tissue processor (Milestone Medical Technologies, Inc, MI, USA)[96]. Paraffin sections were cut with 12μm thickness using a Leica RM2255 microtome (Leica Biosystems Inc. Buffalo Grove, IL, USA) under RNase free conditions. Brains were sectioned coronally through the mesencephalon-diencephalon to allow visualization of the optic tectum and periventricular grey zone, sites showing significant clock gene expression in zebrafish[97] and consistently identifiable despite the small size of the 30dpf midbrain (Fig O in S1 Text). We were unable to consistently section through the same region of the brain in order to compare expression in hypothalamic nuclei due to the size of 30dpf brains [97]. Livers were sectioned longitudinally. Sections were mounted on SureBond charged microscope slides (cat#SL6332-1, Avantik, Springfield, NJ, USA). To allow for comparison between populations, all brains or livers from a single probe set were processed at once (e.g., *rorca-rorcb* brains from all populations, and all time points were processed at the same time). In addition, brains or livers from each time point (CT0, CT8, CT16) were processed on a single slide to allow for a direct comparison between time points within populations.

### RNA FISH, imaging and analysis

RNAscope probes were designed by ACDBio to target all known mRNA transcripts of genes *per1a* (ENSAMXG00000019909), *arntl1a* (ENSAMXG00000011758), *rorca* (ENSAMXG00000009363), and *rorcb* (ENSAMXG00000015029) (Advanced Cell Diagnostics, Hayward, CA, USA) based on Astyanax_mexicanus-2.0, INSDC Assembly (GCA_000372685). Probes were ordered for two-plexing (*per1a-arntl1a* and *rorca-rorcb*) to provide an internal control in each sample for mRNA phase and quantity. Probes may be ordered at the ACD Online Store using the following catalog numbers: *per1a* (590801), *arntl1a* (590831-C2), *rorca* (590811), and *rorcb* (590821-C2). *In situ* hybridization of *per1a*, *arntl1a*, *rorca*, and *rorcb* mRNA was performed on paraffin sections using RNAscope Multiplex Fluorescent Detection Kit v2 (Advanced Cell Diagnostics, Hayward, CA, USA) according to the manufacturer’s protocol.

Slides were stored in the dark at 4°C before imaging. Fluorescent images of sections were taken with a Nikon Eclipse TI equipped with a Yokogawa CSU W1 spinning disk head and Hamamatsu ORCA-Flash 4.O camera for high-resolution imaging using a Nikon 20x/0.75 Plan Apo objective. Probes *rorca* and *per1a* were imaged with 561nm laser and collected with an ET605/70m emission filter, and *arntl1a* and *rorcb* with 633nm laser and collected with an ET700/75m emission filter. DAPI was excited at 405nm and collected with an ET455/50m emission filter. Samples were identified automatically for imaging using a custom script as in Guo *et al*.[98], and each sample was imaged as a tile scan with 10% overlap and z-stack of depth of 18μm with 0.9 μm optical slice thickness. Microscope and camera settings during acquisition were identical across all samples to allow for a direct comparison between timepoints and populations. Image processing in Fiji[99] was identical between populations and time points within a tissue and probe set. Briefly, tiles were stitched into a complete image using Grid/Collection Stitching [100], maximum projected and contrast adjusted. To properly compare cave populations to surface populations, we set the intensity ranges for each image to that of the surface sample images (Fig 4). The intensities of Molino and Tinaja brain images in Fig K in S1 Text are adjusted for oversaturation. Fig 4 excludes the DAPI channel. Corresponding DAPI staining can be found in Fig J in S1 Text. Images are representative of two fish collected from each timepoint per population. For Fig 4, maximum projected images are shown.

For quantification in Fig 5, tiles were stitched into a complete image using Grid/Collection Stitching[100]. Stitched images were sum projected and background subtracted. 400x400 anatomical regions of interest were identified by visual inspection and average intensities were measured for each channel. To allow for a comparison of relative expression per cell from multiple samples, DAPI intensity was measured as a proxy for cell density, and FISH signal intensity was normalized against DAPI intensity for each region. Technical replicates (two to three sections from the same liver or brain) were averaged to make biological replicates represented as points in Fig 5. All groups have two biological replicates with the exception of *rorca*-*rorcb* Pachón brains which have only one due to sample loss. Graphing and statistical analysis were performed using GraphPad Prism software (GraphPad, Prism version 8.3.0, GraphPad Software, San Diego, California USA). For each mRNA probe, cave population means were compared with the control mean (Surface) within timepoints using 2-way ANOVA. Dunnett’s test was used to correct for multiple comparisons across populations and timepoints. Original images underlying this part of the manuscript can be accessed from the Stowers Original Data Repository at ftp://odr.stowers.org/LIBPB-1485.

### Population genomic analyses

Population genetic analyses were performed using samples from Herman *et al*.[50], using individuals from Río Choy (N = 9), Pachón (N = 9 + the reference genome), Tinaja (N = 10), Molino (N = 9), as well as another surface population, Rascón (N = 8). To identify regions that were differentiated between cave and surface populations, we calculated multiple metrics based on the population variant calls (see S1 Text). VCFtools v0.1.13[101] and custom scripts were used to calculate basic population genetic metrics (π, *F*_*ST*_, and *d*_*XY*_) in the coding region per gene. For *F*_*ST*_ and *d*_*XY*_ comparisons, we compared each cave population to two surface populations, Río Choy and Rascón.

To predict the effects of substitutions in circadian genes in cave populations, we employed two *in silico* predictive tools, the Ensembl Variant Effect Predictor (VEP, Ensembl release 100)[102] and Sorting Intolerant from Tolerant (SIFT 4G Annotator, v2.4)[103]. High frequency variants (where a variant is found to be at a frequency of >0.8 in at least one cave population, but not present in the Río Choy surface population) were categorized as potentially deleterious if labelled “high impact” by VEP or “deleterious” by SIFT.

### Testing for relaxed selection on clock genes

Ensembl annotations (v102) were used to identify one-to-one orthologs in teleost lineages and *A*. *mexicanus*. Coding sequences and annotations were downloaded from Ensembl with BioMart. Sequences were aligned with GUIDANCE2’s [104] implementation of the MAFFT algorithm in codon mode [105]. Cavefish sequences were tested for relaxed constraint under the RELAX framework [79]. RELAX fits a null model of 3 ω classes across the phylogeny, and then compares the fit of this to the alternative model, where the branches are subdivided into test and reference sets, with likelihood ratio test (LRT). *k*, defined as the selection intensity parameter, is the exponent of the ω values for the test branches under the alternative model. *k*>1 indicates that selection strength has intensified and *k* < 1 indicates that selection strength has been relaxed. A newick tree with the teleost phylogeny was provided based on the Ensembl Species Tree created built under the Ensembl Compara pipeline. We tested for relaxed selection separately in branches leading to Tinaja/Pachón and Molino based on the relationship between cave and surface populations described in [50]. Further details of this analysis are available in the S1 Text.

### Quantifying melatonin

As in our RNAseq experiment, fish were raised under a 14:10 light-dark cycle. Thirty days post fertilization, melatonin levels were analyzed under (1) light-dark conditions, and (2) under dark-dark conditions, for each population. For dark-dark experiment, fish were kept in total darkness for 24 hours prior to sampling and throughout the duration of the experiment. Ten fish larvae were pooled together, homogenized and melatonin was extracted from each sample as previously described[106] with the following modifications: Methylene chloride was evaporated under a steam of Nitrogen at 40°C. Dried extracts were eluted in 0.7 mL PBS with 0.1% knox gelatin. Each sample was subsequently analyzed using a Direct Saliva Melatonin ELISA (Alpco) following manufacturer’s instructions.

### CRISPR/Cas9 design and genotyping

Functional experiments were performed in CRISPR/Cas9 injected (crispant) larval fish for *aanat2* and *rorca*. CRISPR gRNAs were designed using ChopChop v3 software[107]. gRNAs were designed to target an exon in each of the genes and to produce a double stranded break close to a restriction enzyme site for genotyping purposes. For *aanat2*, the gRNA was 5’-GGTGTGCCGCCGCTGCCGGA-3’ and for *rorca* the gRNA was 5’-gGAGAACGGTAACGGCGGGCA-3’ where the lowercase g at the 5’ end was added to the sequence for T7 transcription (restriction enzyme target sites are underlined). gRNAs were synthesized as previously described[108] with modifications[109,110]. Briefly, gRNA specific oligos were synthesized (IDT) that contained the gRNA target site, T7 promoter, and an overlap sequence:

*aanat2* oligo A:

5’- TAATACGACTCACTATAGGTGTGCCGCCGCTGCCGGAGTTTTAGAGCTAGAAATAGC-3’

*rorca* oligo A:

5’-TAATACGACTCACTATAgGAGAACGGTAACGGCGGGCAGTTTTAGAGCTAGAAATAGC-3’

Each oligo A was annealed to the oligo B:

5’-GATCCGCACCGACTCGGTGCCACTTTTTCAAGTTGATAACGGACTAGCCTTATTTTAACTTGCTATTTCTAGCTCTAAAAC-3’

and these annealed oligos were amplified. gRNAs were transcribed using the T7 Megascript kit (Ambion), as in Klaassen *et al*.[111] and Stahl *et al*.[110], and purified using a miRNeasy mini kit (Qiagen). Nls-Cas9-nls[112] mRNA was transcribed using the mMessage mMachine T3 kit (Life Technologies) following the manufacturer’s instructions and purified using the RNeasy MinElute kit (Qiagen) following manufacturer’s instructions. 150 pg Cas9 mRNA and 25 pg *aanat2* gRNA or 150 pg Cas9 mRNA and 25 pg *rorca* gRNA were injected into single-cell surface fish embryos (2 nL/embryo were injected).

Genomic DNA from injected embryos and wild-type (uninjected) controls was extracted at 48 hours post-fertilization [109,110] and used for genotyping by PCR to determine if mutagenesis was achieved at the locus through gel electrophoresis (Figs R and S in S1 Text). Briefly, gRNAs were designed to disrupt specific restriction enzyme sites; samples were incubated with that restriction enzyme to determine if they contained CRISPR/Cas9 induced mutations that disrupted the restriction enzyme site. The undigested fragment acts as a control while the digested fragment is used to genotype. If the fragment is undigested, this indicates that the restriction enzyme was not able to cut due to the site being disrupted because of the CRISPR/Cas9 induced mutations. Gene specific primers are provided in S1 Data. PCRs were performed with a 56°C annealing temperature and a 1-minute extension time for 35 cycles. The resulting PCR product was split in half, and one half was restriction enzyme digested. A DNA fragment containing the *aanat2* target fragment was digested with *Bb* vI (New England Biolabs Inc.). The *rorca* DNA fragment was digested with *Cac* 8I (New England Biolabs). Both DNA fragments were digested at 37°C for 1 hour.

For wildtype individuals, the PCR fragment was used for cloning. For crispant fish, restriction enzyme digests were performed (as described above) and the undigested bands were gel purified using a gel extraction kit (Qiagen). PCR products were cloned into the pGEM-T Easy Vector (Promega), and three clones per individual were picked, grown, and purified using QIAprep Spin Miniprep Kit (Qiagen) and then sequenced by Sanger sequencing for genotyping (Eurofins Genomics).

### Quantifying sleep behavior

30 dpf injected crispant fish and WT (uninjected) sibling controls were used for all behavioral experiments. Fish were maintained on a 10–14 light-dark cycle throughout development as well as for behavioral experiments. Fish were placed in 12-well tissue culture plates (Cellstar) 18–24 hours before behavioral recording to acclimate to the recording chamber. Fish were fed normal brine shrimp meals before the start of the recording, which began at ZT0 (zeitgeber time) and lasted 24 hours. Video recordings were processed in Ethovision XT (v13). Raw locomotor data was processed with custom-written scripts to quantify sleep duration and behavioral architecture such as locomotor distance, waking activity, sleep bout duration, and sleep bout number[44,83]. For each injected construct, three separate biological replicates were carried out to ensure the phenotype was consistently reproducible. Each biological replicate represented different clutches of fish, injected on different days. Both wildtype and crispant individuals were assessed from each clutch.

## Supporting information

S1 Text Supplemental Methods and Material, Tables A-K, Figs A-W. **Table A.** Numbers of rhythmic genes in each population. **Table B.** Number of genes with loss in rhythmicity (*P* > 0.5) in cave populations compared to rhythmic expression in surface (FDR < 0.1 and FDR < 0.05). **Table C.** Known circadian regulators that are arrhythmic in one or more cave populations. **Table D.** Timing of peak expression of core clock genes (primary and accessory loops) compared between zebrafish and *A*. *mexicanus* populations. **Table E**. Phase shifts between surface and cave populations. **Table F.** The number of significant circadian binding motifs identified in promoter proximal regions of genes with evidence for transcription (*p*<0.05) in the surface population. **Table G.** Arrhythmic genes in cave populations where motif sequences are also lost. **Table H.** Average timing difference in peak expression of circadian feedback loop targets. **Table I**. Number of genes with a significant differential rhythmicity score. **Table J.** Genes associated with GO term DNA-repair are upregulated in cave populations more than expected by chance. *P*-values based on Fisher’s exact tests. **Table K.** *A*. *mexicanus* orthologs of genes that are light induced in zebrafish are not more often upregulated in cavefish compared to surface fish. *P*-values for each surface-cave comparison produced with a hypergeometric test. **Fig A**. Raw reads per sample for Molino. **Fig B.** Raw reads per sample for Pachón. **Fig C.** Raw reads per sample for surface fish. **Fig D.** Raw reads per sample for Tinaja. **Fig E**. A. PC1 and PC2 (explaining 19.1% and 18% of variation, respectively) show that the primary axes of differentiation among samples is ecotype. B. PC3 (explaining 7.1% of variation) separates Molino from other populations. **Fig F.** Cave populations show shifts in phase at *per1a/b* and *cry1a*, *w*ith gene expression peaking later in cave populations compared to the surface population. Expression is represented as normalized read counts. **Fig G.** A-B. In the circles are the circadian phase distributions of predicted targets of RRE and E-BOX for surface fish. Grey bars represent the proportion of each motif seen in each phase. Highlighted in light grey are intervals of phase-specific enrichment for surface fish for each motif. Genes with the RRE motif (C) and EBOX (D) motifs show shifts in the timing of peak expression in cavefish populations. **Fig H.** In the circles are the circadian phase distributions of predicted targets of the D-Box (NFIL3) for surface fish. Grey bars represent the proportion of motifs seen in each phase. Highlighted in light grey is the interval with most significant phase-specific enrichment for surface fish. **Fig I.** Region of interest used in all brain images (white box). Regions include the optic tectum (TeO) and periglomerular grey zone (PGZ) shown in relation to coronal section stained with DAPI. Scale bar is 50μM. **Fig J**. DAPI staining in brain (‘B’, top panels for each timepoint) and liver (‘L’, bottom panels for each timepoint) of surface fish and cavefish (Pachón, Tinaja, Molino) at CT0, CT8, and CT16. A. DAPI channel for sections included in Fig 4A. B. DAPI channels for sections included 4B. **Fig K**. Expression patterns of *per1a* and *arntl1a* in Tinaja and Molino brain images adjusted to correct for oversaturation. **Fig L**. Temporal expression patterns of (A) *per1a* and (B) *arntl1a* in midbrain and liver tissue in *Astyanax mexicanus* populations. *In-situ* staining of *rorca* (A) and *rorcb* (B) using RNAscope in midbrain (‘B’, top panels for each timepoint) and liver (‘L’, bottom panels for each timepoint) of Surface fish and cavefish (Pachón, Tinaja, Molino) at CT0, CT8, and CT16. Each time point is a single fish sample. Images are representative sections of two fish collected per time point, per population. Scale bar is 25μM. **Fig M**. Temporal expression patterns of (A) *rorca* and (B) *rorcb* in midbrain and liver tissue in *Astyanax mexicanus* populations. *In-situ* staining of *rorca* (A) and *rorcb* (B) using RNAscope in midbrain (‘B’, top panels for each timepoint) and liver (‘L’, bottom panels for each timepoint) of Surface fish and cavefish (Pachón, Tinaja, Molino) at CT0, CT8, and CT16. Each time point is a single fish sample. Images are representative sections of two fish collected per time point, per population. Scale bar is 25μM. **Fig N.** *Per1a* expression at each time point measured with RNAseq in whole fry and in the brain and liver with RNA FISH. Grey dotted lines represent a loess regression for visualization purposes. **Fig O.** *Arntl1a* expression at each time point measured with RNAseq in whole fry and in the brain and liver with RNA FISH. Grey dotted lines represent a loess regression for visualization purposes. **Fig P.** *Rorca* expression at each time point measured with RNAseq in whole fry and in the brain and liver with RNA FISH. Grey dotted lines represent a loess regression for visualization purposes. **Fig Q.** *Rorcb* expression at each time point measured with RNAseq in whole fry and in the brain and liver with RNA FISH. Grey dotted lines represent a loess regression for visualization purposes. **Fig R.** Analysis of mutagenesis in *aanat2* crispant F_0_ fish. **A**. Genotyping gel of uninjected control and injected embryos. A portion of *aanat2* genomic region was amplified by PCR from DNA extracted from individual embryos. Labeled D is half of the PCR product that was digested with BbvI. Unlabeled is undigested PCR product. Indels can disrupt the restriction enzyme site, leading to undigested PCR product in injected embryos. **B**. Diagram of *aanat2* gene based on the surface fish reference genome (Ensembl v98). Boxes indicate exon and lines indicate introns. The empty boxes are 5’ and 3’ UTR and the closed boxes are coding sequence. A gRNA was designed targeting exon 1. The gRNA target site is in blue and the PAM sequence is in red. The underlined sequence is the BbvI restriction enzyme recognition sequence used for genotyping. The arrow indicates the predicted Cas9 cut site. Gene structure was generated using http://wormweb.org/exonintron and then modified. **C**. Sequence of wildtype surface fish and sequence of six clones from the restriction enzyme resistant band from *aanat2* injected individuals. The total number of base pairs less than the wildtype sequence is indicated to the right of each clone. **Fig S.** Analysis of mutagenesis in *rorca* crispant F_0_ fish. A. Genotyping gel of uninjected control and injected embryos. A portion of *rorca* genomic region was amplified by PCR from DNA extracted from individual embryos. Labeled D is half of the PCR product that was digested with Cac8I. B. Diagram of *rorca* gene based on the Pachón Ensembl v93 genome. Boxes indicate exon and lines indicate introns. The empty boxes are UTR and the closed boxes are coding sequence. A gRNA was designed targeting exon 6. The gRNA target site is in blue and the PAM site is in red. The underlined sequence is the Cac8I restriction enzyme recognition sequence used for genotyping. The arrow indicates the predicted Cas9 cut site. Gene structure was generated using http://wormweb.org/exonintron and then modified. C. Sequence of wildtype surface fish and sequence of 3 clones from the restriction enzyme resistant band from *rorca* injected individuals. The total number of base pairs more or less than the wildtype sequence is indicated to the right of each clone. **Fig T.** A. Expression of *per2*, a light-activated clock gene, over the course of the day in surface and cave populations (JTK_cycle *p*-values: surface, *p =* 0.09, *q* = 0.7; Molino, *p* = 0.07, *q* = 1; Pachón, *p* = 0.003; *q* = 0.16; Tinaja, *p* = 0.016; *q* = 0.22). Rhythmicity was not found to be different between populations (all comparisons, S_DR_ *p*>0.68, *q* = 1). B. Base level expression of *per2* between populations. *Per2* has lower base level expression in the surface than Pachón (log_2_-fold change = 0.82) and Tinaja (log_2_-fold change = 0.56), but higher expression than Molino (log_2_-fold change = 1.3). **Fig U.** Core circadian genes and melatonin regulator *aanat2* show differentiated rhythmicity from surface fish in at least one cave population. **Fig V.** *Exo-rhodopsin* has robust rhythmic expression in the surface population (*p* = 9.26 x 10^−5^, *q* = 0.006), but is not strongly in rhythmic in cave populations (Pachón, *p* = 0.04, *q* = 0.32; Tinaja, *p* = 1, *q* = 1.0; Molino, *p* = 0.37, *q* = 1.0). **Fig W.** Phylogenetic tree of species used for RELAX analysis for changes in selection intensity. Black branches indicate “reference” branches, where blue branches (cavefish lineages of *A*. *mexicanus*) have been used as foreground branches to test for changes in selection intensity.
(PDF)

S1 Data Supplemental raw data. (XLSB)

S2 Data Raw VEP output. (ZIP)

S3 Data Raw SIFT output. (XLSX)
