# Supplementary material for: Repeated evolution of circadian clock dysregulation in cavefish populations
Source: PLoS Genet. 2021 Jul 12;17(7):e1009642. doi: 10.1371/journal.pgen.1009642 (PMC8297936; doi:10.1371/journal.pgen.1009642)

Supplemental Material

S1 Appendix: *Supplemental Methods and Material*…………………..……………………..…...2-7
Supplemental References…………………………………………...…….……………………..8-19
Tables **A-K**……………………………………..………………..……………..……10-21
Figs **S1-S23**……..……………………………………….………...……...……….22-45

S1 Data: Supplemental raw data

1. Number of uniquely mapped reads per sample and sample annotations (population, timepoint)
2. JTK_cycle results for each population
3. Genes that are rhythmic in surface but arrhythmic in at least 1 cave (p>0.5)
4. Curated list of genes with orthologs annotated as involved in circadian rhythm with their JTK_cycle p-values in cave and surface populations
5. Pairwise S_{DR} p-values between cave and surface populations
6. Phases called by JTK_cycle for genes across populations (includes genes without significant rhythmicity)
7. Raw read counts for each sample
8. Primer sequences
9. Genetic differentiation at genes with changes in rhythmicity between surface and cave populations
10. Pairwise comparisons of basal expression between surface and cave populations across timepoints with DESeq2
11. Gene Ontology terms associated with cycling genes (FDR<0.05 in JTK_cycle) in each population
12. Genes with improved rhythmicity in cavefish populations
13. . JTK p-values for DNA repair genes
14. GO term enrichment for arrhythmic genes shared across caves that are rhythmic in the surface.
15. RELAX results

S2 Data: Raw VEP output

S3 Data: Raw SIFT output

Supplemental Methods and Material

Sampling

Fry used in this experiment are derived from lab born individuals from 3 cave populations (Molino, Pachón, and Tinaja) and one surface population (Río Choy). Individuals from these populations have been maintained in the lab for approximately 15 years and individuals in used in these experiments are estimated to be a few generations removed from the wild. The Pachón and Tinaja population are sister taxa, as are Molino and Río Choy. Previous population genetic analyses of individuals from these localities indicate that individuals from each population cluster together, indicating that they are discrete populations, though there is also a history of admixture[1].

All samples from each population were derived from the same mating clutch and raised in 14:10 light-dark cycle. All fish were exactly 30 days post fertilization at the start of sampling. Fish were kept in total darkness for 24 hours prior to sampling and throughout the duration of the experiment. Fish were first sampled at 6am (Circadian Time 0) and then every four hours throughout until 2am (corresponding to CT 20). We cannot exclude the possibility fluctuations in gene expression are still affected by previous light-dark conditions. However, as cave and surface populations are raised under identical light regimes, this should have not impact inferences of evolved differences in the biological clock based on expression comparisons between cave and surface fish. Sufficient food was in tanks so that fish could eat *ad libitum*. Prior to the experiment days, additional food was added within a 2-3hr window. During the experiment itself, food was added twice daily, in the morning and evening (exactly at 8am and 8pm).

Six individuals were flash frozen separately for every time point and population and stored at -80°C until RNA was extracted.

RNA extraction and library preparation

For RNA isolation, all individuals were processed within a week of each other (between 1-19-2017 and 1-24-2017). Whole organisms (< 30 mg of tissue) were homogenized using Fisher brand pellet pestles and cordless motor (Fisher Scientific) in the lysate buffer RLT plus. Total RNA was extracted using the Qiagen RNAeasy Plus Mini Kit. Extraction batch was randomized across populations and treatments.

All cDNA libraries were constructed at the University of Minnesota Genomics Center on the same day in the same batch. In brief, a total of 400 ng of RNA was used to isolate mRNA via oligo-dT purification. dsDNA was constructed from the mRNA by random-primed reverse transcription and second-strand cDNA synthesis. Strand-specific cDNA libraries were then constructed using TruSeq Nano Stranded RNA kit (Illumina), following the manufacturer's protocol. Library quality was assessed using Agilent DNA 1000 assay on a Bioanalyzer. To minimize sequencing lane effects, barcoded libraries were pooled with treatment and population spread evenly between lanes. Samples were sequenced across multiple lanes of an Illumina HiSeq 2500 to produce 125-bp paired-end reads at University of Minnesota Genomics Center. All sequence data were deposited in the short-read archive (SRA).

Read mapping and normalization

Raw reads from each sample were assessed with Fastqc (v0.11.7), then cleaned of adapter contamination and low-quality bases with Trimmomatic (v0.33)[2]. Cleaned reads were mapped to the *Astyanax mexicanus* draft genome assembly version 1.02 (GCA_000372685.1) with STAR[3]. Counts for each gene were generated with stringtie (v1.3.3d)[4] based on the *Astyanax mexicanus* Ensembl v91 annotation. The Python script prepDE.py, bundled with stringtie, was used to generate a counts matrix for filtering, normalization, and subsequent analysis with DESeq2[5].

Genes with less than 100 total counts across all samples in the experiment were removed from the analysis. Counts were then normalized by sample library size and transformed with a variance stabilizing transformation with DESeq2. The variance stabilizing transformation makes the normalized counts values have approximately constant variance across mean expression values, which reduces bias due to highly expressed genes having greater variance than lowly expressed genes. Variance-stabilized counts were then decomposed for a principal component analysis (PCA) of all samples to identify broad patterns of differentiation among populations and timepoints. Principal components analysis was performed with the 'prcomp' package in the R computing environment.

Promoter analysis of rhythmic genes

Putative promoter sequences (1-kb upstream to 200bp downstream from the transcription start site (TSS)) were extracted based on the *A. mexicanus* genome assembly using samtools faidx. As the draft *A. mexicanus* genome assembly (v1.02) was built from an individual of Pachón cave descent, we used SNP and indel calls from population data[1] to create alternative reference sequences for Río Choy and cave populations by inserting variant calls into each reference sequence (see "Variant calling and population genetics measures" below). Variants where all genotyped individuals were homozygous for the alternative allele and at least 5 of 9 individuals were genotyped were inserted into the reference. LiftOver coordinates were created between alternative references to compare sequences between populations. When promoter regions, annotated based on the Pachón cave reference genome, were not available in an alternative population reference, the regions were not compared between surface-cave pairs. FIMO of the MEME suite[6] was then used identify motifs in each promoter. We used the default *p*-value cut-off of < 0.0001 of FIMO to identify significant motifs in each putative promoter sequence.

To address whether the loss of cycling at ancestrally rhythmic genes in cave populations is a consequence of disruptions to transcription factor binding motifs, we tested whether genes rhythmic in surface fish are differentially enriched for transcription factor binding motifs between cave and surface fish with HOMER[7]. As described above, we created putative promoter sequences for each population, and then tested whether promoters at genes rhythmic in surface fish (where $p<0.05$) are enriched for motif sequences compared to the same sequences in each cavefish population. The HOMER motif library, including known circadian motifs, was used for motif enrichment testing. Each cave-surface comparison yielded no significant enrichment of motifs in the surface fish population.

Phase enrichments of putative clock targets in cavefish

After identifying putative clock targets, we compared the phase of putative clock targets in surface fish and cavefish. Consistent with the phase shifts seen in the core clock (Table D), putative clock targets also show shifts in their phase in cavefish compared to surface fish (Table H, Fig. G). In the case of the E-box, in cave populations the phase intervals with the highest proportion of these motifs occur later (CT 4, 6, 6-10 in Molino, Pachón, and Tinaja, respectively) than what is seen in the surface population (CT 2) (Fig. G). In contrast, for the RRE and D-box motif, there is no phase with enrichment in cave populations.

Variant calling and population genetics measures

Population genetic analyses were performed using samples from Herman *et al.*[1], using individuals from Río Choy (N=9), Pachón (N=9 + the reference genome), Tinaja (N=10), Molino (N=9), as well as another surface population, Rascón (N=8). In brief, variant calling was performed using the Genome Analysis Toolkit v3.3.0 (GATK) based on GATK Best Practices[8,9]. Duplicate reads were marked using Picard's MarkDuplicates tool and filtered from downstream analyses resulting in a mean coverage of 9.28x. GATK's IndelRealigner (IR) and RealignerTargetCreator (RTC) were used to realign reads that may have been misaligned around insertions/deletions. HaplotypeCaller and then GenotypeGVCFs were then used to generate variant calls for all individuals. Low confidence variants were filtered using the VariantFiltration and SelectVariants tools.

To identify regions that were differentiated between cave and surface populations, we calculated multiple metrics based on the population variant calls. For population genomic metrics, we included the samples described above, as well as the Pachón reference genome[10], and required six or more individuals have data for a particular site. We excluded masked repetitive elements, indels, and the 10-bp surrounding indels. VCFtools v0.1.13[11] and custom scripts were used to calculate basic population genetic metrics (π, F_{ST}, and d_{XY}) in the coding region per gene. For F_{ST} and d_{XY} comparisons, we compared each cave population to two surface populations, Río Choy and Rascón.

Genes were considered F_{ST} outliers if the average F_{ST} of a gene was in the largest 5% of genes in the genome for a surface-cave comparisons (e.g., surface Río Choy vs. Pachón cave). As relative measures of divergence like F_{ST} can be driven by low diversity due to low recombination or other genomic features[12], we also considered average d_{XY}. We considered genes as having a high d_{XY} if average d_{XY} was within the largest 20% of values for all genes in the genome for the focal comparison.

HapFLK v1.3[13] was used to estimate the HapFLK statistic for *Astyanax mexicanus* samples and two *Astyanax aeneus* samples sequenced in Herman *et al.*[1]. The Texas surface *Astyanax mexicanus* was excluded, as no population-level sampling was conducted for this population. HapFLK accounts for hierarchical population structure by building local ancestry and detecting changes in haplotype frequencies which exceed what is expected for selectively neutral evolution[13]. This method is thought to be robust to bottlenecks and migration and has performed well in comparison to other statistical tests for selection[14]. A population-specific tree was created for each scaffold with HapFLK prior to tests for selection. Reynolds distances and a kinship matrix were calculated with HapFLK. HapFLK was subsequently run on unphased data with 5 clusters (-K 5) and 20 EM runs to fit the LD model (-nfit = 20). *P*-values were

estimated by fitting a standard normal distribution genome-wide. Genes with at least one *p*-value < 0.05 within the gene were subsequently compared to cave-surface F_{ST} outliers to identify putative evidence for selection on the circadian transcriptome. Outside of the core clock, we found that seven genes in this set of F_{ST} outliers also showed signatures of selection based on the HapFLK statistic, which detects changes in haplotype frequencies which exceed what is expected under neutral evolution[13] (see Supplemental Methods).

SIFT and VEP

To predict the effects of substitutions in circadian genes in cave populations, we employed two *in silico* predictive tools, the Ensembl Variant Effect Predictor (VEP, Ensembl release 100)[15] and Sorting Intolerant from Tolerant (SIFT 4G Annotator, v2.4)[16]. All predictions were performed on the population samples described above, mapped to the surface fish genome assembly (Astyanax_mexicanus-2.0 assembly). Variant calling was performed as described above.

VEP was used to identify variants with predicted deleterious impacts on protein function. VEP provides a rating based on predicted impact, where "high" impact mutations are predicted to have a disruptive impact in the protein (i.e., by causing protein truncation, loss of function, or resulting in nonsense mediated decay).

We predicted the consequences of amino acid changing variants with SIFT, which uses sequence homology to predict the impact of nonsynonymous substitutions. To run SIFT, we created a local SIFT 4G database for *Astyanax mexicanus* based on the Astyanax_mexicanus-2.0 assembly. An amino acid substitution is predicted to be damaging if the score is <= 0.05, and tolerated if the score is > 0.05.

High frequency variants (where a variant is found to be at a frequency of >0.8 in at least one cave population, but not present in either the Río Choy or Rascon surface population) were categorized as potentially deleterious if labelled "high impact" by VEP or "deleterious" by SIFT.

Testing for changes in selection intensity

Ensembl annotations were used to identify one-to-one orthologs between clock genes in *A. mexicanus* and other teleost lineages (zebrafish, stickleback, Red-bellied piranha, Channel catfish, electric eel, Brown trout, Rainbow trout, Atlantic salmon, Pinecone Soldierfish, Channel Bull Blenny, Asian bonytongue, Atlantic herring, Huchen, Northern Pike, Yellowtail amberjack, Nile Tilapia, Cod, Denticle herring, Amazon molly, Fugu, Tetradon, Japanese Medaka, Common Carp)(Fig W). One-to-one orthologs with *A. mexicanus* sequences were not available for all reference genomes surveyed). Coding sequences were downloaded from Ensembl from all species using Ensembl Biomart. When multiple CDS sequences were available for a given gene, the longest sequence was chosen for alignment. One-to-one orthologs of coding sequences were aligned using the GUIDANCE2's implementation of the MAFFT algorithm in codon mode. Alignments were then visually inspected for issues. Coding sequences for the Pachón cave population were derived from the Pachón reference genome and associated annotations (Ensembl v102); coding sequences for Tinaja and Molino were created by inserting variant calls from a randomly selected cave individual[1] into the *A. mexicanus* reference genome to create

alternative pseudo-reference genomes. Further details on variant calling are described above (see "***Variant calling and population genetics measures***").

RELAX[17], implemented in the hyphy 2.3.6 package, was used to test for relaxed or insensitive selection strength in cavefish lineages. A species phylogeny was provided for each test based on the Ensembl species tree. RELAX fits a null model of 3 ω classes, comparing the fit of this to the alternative model where branches are divided into "test" (i.e., cavefish branches) and "reference" (i.e., all other branches) with an LRT. As cave forms are not monophyletic[1] and different evolutionary forces may be at play in different lineages, branches leading to Pachón/Tinaja and Molino were assessed separately as "test" branches versus all other branches as background.

Overlap with rhythmic transcripts in zebrafish

To compare rhythmic transcripts in *A. mexicanus* with those of zebrafish, we compared genes with a JTK_cycle p-value < 0.05 to genes annotated as cycling in zebrafish in previous studies[18,19]. Genes annotated as significantly cycling under dark-dark and light-dark conditions in zebrafish larvae[18] and cycling in zebrafish liver under light-dark conditions[19] were downloaded and annotated to 1-to-1 orthologs of *A. mexicanus* genes for comparison.

Annotating known circadian genes

To create a list of known circadian genes, we downloaded 1-to-1 orthologs with zebrafish that were associated with the Go Ontology term "Circadian Rhythm." This list was also supplemented with genes from the literature[18]. The full list is found in S1 Data (see tab "KnownCircadianPvalues"). Notably, three genes involved in the core feedback loop were not found to cycle significantly (at FDR<0.1) in any population: *clocka*, *clockb*, and *per2*. *Per2* is light activated and does not cycle robustly under darkness in zebrafish[18,20] (FigFig P). *Clocka/b* were lowly expressed in our dataset (average of 2.36 reads/sample and 12.8 reads/sample and for *clocka* and *clockb*, respectively), so we may lack power to infer rhythmicity at these genes.

Identifying bases shifts between populations

To identify base shifts (i.e., change in levels of expression but not necessarily a change in rhythm), we treated all timepoints as replicates and tested for differential expression with DESeq2 between pairs of populations (See S1 Data, tab "Baseshifts"). DESeq2 was used to normalize raw gene read counts, estimate dispersion factors for each gene, and then test for differential expression based on a negative binomial distribution with the default correction for multiple testing. To call differential expression, we retained genes at a cut-off of $p_{adj} = 0.05$.

Upregulation of DNA repair genes in cavefish populations

Environmental light plays an crucial role in regulating DNA damage responses and cell cycle control in teleosts[21,22]. Previous work in the *A. mexicanus* system has found that cave populations show lower levels of DNA damage and increased expression of the DNA repair genes *CPD photolyas*e (*CPD phr*) and *damage-specific DNA binding protein 2* (*ddb2*).[23] To explore this in our dataset, we ask whether genes associated with the GO term for DNA repair (GO:0006281) showed higher basal expression in cave populations compared to the surface population in dark-dark conditions. We found that DNA-repair associated genes are upregulated

in cave populations more than expected by chance (Fisher's exact tests, Table J, all *p*-values < 0.0003).

To ask whether this class of genes has also undergone changes in temporal dynamics as well as base expression levels, we next compared rhythmic expression across this group of genes by comparing the distribution of JTK_cycle *p*-values between cave and surface pairs for DNA repair genes (see S1 Data). We found that DNA repair genes show a shift towards higher *p*-values in Molino (Wilcoxon signed rank test, $p = 0.002$) compared to the surface population, suggesting a shift towards less rhythmic expression for DNA repair genes in the Molino population. Conversely, we found no difference between the surface population and Tinaja and Pachón populations (Wilcoxon signed rank test, $p = 0.95$ and $p = 0.58$, respectively). However, while we do not find increased rhythmicity across DNA-repair genes overall in Pachón, we did find that genes that were uniquely rhythmic in Pachón (e.g., only cycle in Pachón and no other population) were enriched for the GO term DNA repair (9 genes, 6.42-fold enrichment, q=1.15E-02), as well as the related term "cellular response to DNA damage stimulus" (10 genes, 5.66-fold enrichment, q=1.22E-02).

Comparison of basal expression of light-activated genes

Previous work has shown that the light-activated gene *per2* is upregulated in fin clips of cavefish from the Pachón and Chica caves[23]. The upregulation of this light-activated gene in cavefish has been suggested as evidence for constitutive upregulation of cavefish clock, similar to the condition of being under constant light[23]. To explore this question, we surveyed *A. mexicanus* orthologs of light-activated genes in zebrafish[18]. Light-activated genes are activated under the light phase in light-dark conditions, but maintain low levels of expression under dark-dark conditions[18]. Comparing basal levels of expression of this gene set across populations, we did not find that genes associated with light induction were more likely to be upregulated in cavefish compared to surface fish (hypergeometric tests, all p>0.84, Table K). Nor do we find that light-activated genes in the core circadian feedback loop are consistently upregulated in cave populations. *Per2* showed higher expression in Tinaja (q=0.07) and Pachón (q=0.02), but was downregulated in Molino compared to the surface (q=0.004)(Fig T). The light-activated gene *cry1aa*, which plays a key role in light entrainment[24], did not show differences in basal expression between cave and surface populations in our analysis (Tinaja, q=0.68; Molino, q=0.79; Pachón, q=0.82). Other cryptochromes which have been found to be light induced (*cry2a*, *cry3*)[25] also did not show differences in basal expression between cave and surface populations.

Animal care and use

Procedures for all experiments performed at Florida Atlantic University were approved by the Institutional Animal Care and Use Committee at Florida Atlantic University (Protocols #A15-32 and #A18-38). Experiments performed at the Stowers Institute for Medical Research was approved by the Institutional Animal Care and Use Committee (IACUC) of the Stowers Institute for Medical Research. NR's institutional authorization for use of *A. mexicanus* in research is 2019-084.

Supplemental References

1. Herman A, Brandvain Y, Weagley J, Jeffery WR, Keene AC, Kono TJY, et al. The role of gene flow in rapid and repeated evolution of cave-related traits in Mexican tetra, Astyanax mexicanus. Molecular Ecology. 2018;27: 4397–4416. doi:10.1111/mec.14877

2. Bolger AM, Lohse M, Usadel B. Trimmomatic: a flexible trimmer for Illumina sequence data. Bioinformatics. 2014;30: 2114–2120. doi:10.1093/bioinformatics/btu170

3. Dobin A, Davis CA, Schlesinger F, Drenkow J, Zaleski C, Jha S, et al. STAR: ultrafast universal RNA-seq aligner. Bioinformatics. 2013;29: 15–21. doi:10.1093/bioinformatics/bts635

4. Pertea M, Kim D, Pertea GM, Leek JT, Salzberg SL. Transcript-level expression analysis of RNA-seq experiments with HISAT, StringTie and Ballgown. Nature Protocols. 2016;11: 1650–1667. doi:10.1038/nprot.2016.095

5. Love MI, Huber W, Anders S. Moderated estimation of fold change and dispersion for RNA-seq data with DESeq2. Genome Biology. 2014;15: 550. doi:10.1186/s13059-014-0550-8

6. Bailey TL, Johnson J, Grant CE, Noble WS. The MEME Suite. Nucleic Acids Res. 2015;43: W39–W49. doi:10.1093/nar/gkv416

7. Heinz S, Benner C, Spann N, Bertolino E, Lin YC, Laslo P, et al. Simple combinations of lineage-determining transcription factors prime cis-regulatory elements required for macrophage and B cell identities. Mol Cell. 2010;38: 576–589. doi:10.1016/j.molcel.2010.05.004

8. McKenna A, Hanna M, Banks E, Sivachenko A, Cibulskis K, Kernytsky A, et al. The Genome Analysis Toolkit: A MapReduce framework for analyzing next-generation DNA sequencing data. Genome Res. 2010;20: 1297–1303. doi:10.1101/gr.107524.110

9. DePristo MA, Banks E, Poplin RE, Garimella KV, Maguire JR, Hartl C, et al. A framework for variation discovery and genotyping using next-generation DNA sequencing data. Nat Genet. 2011;43: 491–498. doi:10.1038/ng.806

10. McGaugh SE, Gross JB, Aken B, Blin M, Borowsky R, Chalopin D, et al. The cavefish genome reveals candidate genes for eye loss. Nat Commun. 2014;5: 1–10. doi:10.1038/ncomms6307

11. Danecek P, Auton A, Abecasis G, Albers CA, Banks E, DePristo MA, et al. The variant call format and VCFtools. Bioinformatics. 2011;27: 2156–2158. doi:10.1093/bioinformatics/btr330

12. Cruickshank TE, Hahn MW. Reanalysis suggests that genomic islands of speciation are due to reduced diversity, not reduced gene flow. Molecular Ecology. 2014;23: 3133–3157. doi:10.1111/mec.12796

13. Fariello MI, Boitard S, Naya H, SanCristobal M, Servin B. Detecting signatures of selection through haplotype differentiation among hierarchically structured populations. Genetics. 2013;193: 929–941. doi:10.1534/genetics.112.147231

14. Schlamp F, Made J van der, Stambler R, Chesebrough L, Boyko AR, Messer PW. Evaluating the performance of selection scans to detect selective sweeps in domestic dogs. Molecular Ecology. 2016;25: 342–356. doi:10.1111/mec.13485

15. McLaren W, Gil L, Hunt SE, Riat HS, Ritchie GRS, Thormann A, et al. The Ensembl Variant Effect Predictor. Genome Biol. 2016;17: 122. doi:10.1186/s13059-016-0974-4

16. Ng PC, Henikoff S. SIFT: predicting amino acid changes that affect protein function. Nucleic Acids Res. 2003;31: 3812–3814. doi:10.1093/nar/gkg509

17. Wertheim JO, Murrell B, Smith MD, Kosakovsky Pond SL, Scheffler K. RELAX: Detecting Relaxed Selection in a Phylogenetic Framework. Molecular Biology and Evolution. 2015;32: 820–832. doi:10.1093/molbev/msu400

18. Li Y, Li G, Wang H, Du J, Yan J. Analysis of a gene regulatory cascade mediating circadian rhythm in zebrafish. PLOS Computational Biology. 2013;9: e1002940. doi:10.1371/journal.pcbi.1002940

19. Boyle G, Richter K, Priest HD, Traver D, Mockler TC, Chang JT, et al. Comparative analysis of vertebrate diurnal/circadian transcriptomes. PLOS ONE. 2017;12: e0169923. doi:10.1371/journal.pone.0169923

20. Vatine G, Vallone D, Gothilf Y, Foulkes NS. It's time to swim! Zebrafish and the circadian clock. FEBS Letters. 2011;585: 1485–1494. doi:10.1016/j.febslet.2011.04.007

21. Dekens MPS, Santoriello C, Vallone D, Grassi G, Whitmore D, Foulkes NS. Light regulates the cell cycle in zebrafish. Current Biology. 2003;13: 2051–2057. doi:10.1016/j.cub.2003.10.022

22. Tamai TK, Vardhanabhuti V, Foulkes NS, Whitmore D. Early embryonic light detection improves survival. Current Biology. 2004;14: R104–R105. doi:10.1016/j.cub.2004.01.014

23. Beale A, Guibal C, Tamai TK, Klotz L, Cowen S, Peyric E, et al. Circadian rhythms in Mexican blind cavefish Astyanax mexicanus in the lab and in the field. Nat Commun. 2013;4: 1–10. doi:10.1038/ncomms3769

24. Tamai TK, Young LC, Whitmore D. Light signaling to the zebrafish circadian clock by Cryptochrome 1a. Proc Natl Acad Sci U S A. 2007;104: 14712–14717. doi:10.1073/pnas.0704588104

25. Weger BD, Sahinbas M, Otto GW, Mracek P, Armant O, Dolle D, et al. The light responsive transcriptome of the zebrafish: function and regulation. PLOS ONE. 2011;6: e17080. doi:10.1371/journal.pone.0017080

Table A. Numbers of rhythmic genes in each population

	Number of rhythmic genes	
	FDR < 0.05	FDR < 0.1
Surface	539	768
Tinaja	327	616
Molino	83	106
Pachón	88	193

Table B. Number of genes with loss in rhythmicity ($P > 0.5$) in cave populations compared to rhythmic expression in surface (FDR < 0.1 and FDR < 0.05). 539 genes were rhythmic in the surface population at FDR < 0.05, and 768 genes were rhythmic in the surface population at FDR < 0.1.

	0.1 FDR	0.05 FDR
Tinaja	391	289
Molino	397	266
Pachón	393	252

Table C. Known circadian regulators that are arrhythmic in one or more cave populations

Gene	Arrhythmic
Nfil3	Pachón
Aanat2	Tinaja, Molino
Cry1ba	Tinaja, Pachón
Cry1bb	Tinaja, Pachón
Arntl2	Tinaja, Molino, Pachón
Cry4	Pachón
Nptx2b	Pachón, Molino

Table D. Timing of peak expression of core clock genes (primary and accessory loops) compared between zebrafish and *A. mexicanus* populations.

	Zebrafish	Surface	Tinaja	Molino	Pachón
Arntl1a	14.5	14	22	16	20
Arntl1b	13.8	14	20	16	16
Arntl2	17.4	18	8	22	N/A[1]
Per1a	2.3	2	6	6	6
Per1b	4.5	4	8	6	8
Rorca	12	12	14	12	10
Rorcb	13.3	12	14	16	14
Cry1ab	7.6	10	N/A[1]	8	0
Bhlhe40	5.3	4	8	8	8
Bhlhe41	1.3	0	6	2	4
Nfil3	16.2	12	2	14	4
Cry4	15.3	16	20	22	22
Cry1bb	15.4	18	2	18	0
Cry1ba	14.3	16	N/A[1]	20	N/A[1]
Nr1d1	0.8	0	6	2	4

[1]N/A indicates that amplitude is estimated at 0

Table E. Phase shifts between surface and cave populations

	Average shift	*P-value*
Surface-Pachón	2.03	$< 2.2 \times 10^{-16}$
Surface-Tinaja	1.3	$< 2.2 \times 10^{-16}$
Surface-Molino	0.48	$< 2.2 \times 10^{-16}$

Table F. The number of significant circadian binding motifs identified in promoter proximal regions of genes with evidence for transcription ($p<0.05$) in the surface population.

	Identified sequences	Number of genes
E-box(Arntl)	426	296
D-box(NFIL3)	1240	602
RORC	776	775

Table G. Arrhythmic genes in cave populations where motif sequences are also lost.

Gene	Motif	Motif *p*-value in surface fish[1]	Matched sequence in surface fish[1,2]	Cave population in which motif is lost
ENSAMXG00000015742	D-box(NFIL3)	8.81E-05	TTTTGTAATCT	Molino
yme1l1a	D-box(NFIL3)	3.73E-05	TTATGTAAGTG	Molino
gys1	D-box(NFIL3)	7.81E-05	TTATATAATTT	Molino
hdac1	D-box(NFIL3)	8.88E-05	TTATATAATAA	Molino
si:dkey-32e6.3	D-box(NFIL3)	3.9e-05;7.81e-05	TTATATAACCT;TTATATAATTT	Molino
edem3	D-box(NFIL3)	4.26E-05	TTATATAATGT	Molino
rab1ab	D-box(NFIL3)	3.91E-06	TTATGTAATAT	Molino
kdelr3	D-box(NFIL3)	4.01E-05	TGATGTAACTT	Molino
dhrs13a.2	D-box(NFIL3)	1.62e-05;3.02e-05	TTACGTAACCA;TTACGTAAGCC	Molino
ENSAMXG00000027572	D-box(NFIL3)	4.8e-05;9.19e-05	TTATATAATAT;TTATATAACTA	Molino
ccdc187	D-box(NFIL3)	1.83e-05;2.92e-05	TTACGTAACAG;TTACGTAAGTT	Molino
si:ch211-191i18.4	D-box(NFIL3)	1.22e-05;8.2e-05	TTATGTAATTT;TTACATAACAA	Molino
si:ch211-157p22.10	D-box(NFIL3)	2.47E-05	TTATGTAAGAA	Molino
HOXC4	D-box(NFIL3)	9.86E-05	ATATGTAATGC	Molino
gata4	D-box(NFIL3)	1.83e-05;2.75e-05	TTACGTAATAC;TTACGTAAGAC	Molino
si:dkey-32e6.3	D-box(NFIL3)	3.9e-05;7.81e-05	TTATATAACCT;TTATATAATTT	Tinaja
ENSAMXG00000001997	D-box(NFIL3)	6.49e-06;1.83e-05	TTACGTAATGT;TTACGTAATAC	Pachón
ptges3a	D-box(NFIL3)	7.65E-05	ATATGTAATAT	Pachón
mapkapk2a	D-box(NFIL3)	8.97E-05	TTTTGTAACAA	Pachon
cry1bb	D-box(NFIL3)	4.80E-05	TTATATAATAT	Pachón
ENSAMXG00000019162	D-box(NFIL3)	2.58E-05	TTATGTAATTA	Pachón
si:dkey-17e16.10	D-box(NFIL3)	4.26E-05	TTATATAATGT	Pachón
mcm4	E-box(Arntl)	1.28E-05	AGTCACGTGG	Molino
ENSAMXG00000008961	E-box(Arntl)	3.48E-05	CCTCACGTGT	Molino
pard6ga	E-box(Arntl)	1.28E-05	AGTCACGTGG	Molino
ENSAMXG00000020281	E-box(Arntl)	5.87e-05;6.17e-05	TAGCACGTGC;AAGCACGTGC	Molino
gramd4b	E-box(Arntl)	4.63E-05	CTTCACGTGC	Molino
atad1b	E-box(Arntl)	3.95E-05	TCTCACGTGT	Pachón
tent5ba	E-box(Arntl)	2.41E-05	AATCACGTGT	Pachón
btr01	RORC	6.40E-05	AAAACTGGGTTA	Molino
ENSAMXG00000008655	RORC	8.49E-05	GAAAATGGGTTA	Molino
fdxacb1	RORC	5.85E-05	TTAAACAGGTCA	Molino
dpf3	RORC	1.87E-05	AAAATTAGGGCA	Molino
ENSAMXG00000009628	RORC	2.64E-05	ATAAGCAGGTCA	Molino
PAFAH1B2	RORC	8.72E-05	GAAAGTATGTCA	Molino
col8a1a	RORC	3.85E-06	AAAAGTGGGTCA	Molino

rpl7l1	RORC	3.76E-05	AAAATGAGGTCA	Molino
zgc:92040	RORC	3.70E-05	AATAGTAGGTCA	Molino
SLC25A38	RORC	4.11E-05	ATTATTAGGTCA	Molino
si:dkey-275b16.2	RORC	9.88E-05	ATAACTAGGTCT	Molino
hyal2b	RORC	9.39E-05	TATTATAGGTCA	Tinaja

[1]The motif sequence identified in the surface fish promoter

[2]When multiple motifs were identified in a proximal promoter, respective sequences and *p*-values are separated by a semi-colon.

TableH. Average timing difference in peak expression of circadian feedback loop targets

	Average difference in peak expression
	E-BOX (Arntl)
Molino-surface	3.4
Pachón-surface	5.52
Tinaja-surface	7.24
	RRE (Rorc)
Molino-surface	4.4
Pachón-surface	5
Tinaja-surface	7
	D-BOX (NFIL3)
Molino-surface	4.11
Pachón-surface	5.23
Tinaja-surface	6.8

Table I. Number of genes with a significant differential rhythmicity score

	Number of differentially rhythmic genes (FDR<0.1)
surface vs. Molino	148
surface vs. Tinaja	174
surface vs. Pachón	185

Table J. Genes associated with GO term DNA-repair are upregulated in cave populations more than expected by chance. *P*-values based on Fisher's exact tests.

	DNA-repair	Not DNA-repair	*p*-value
Upregulated in Molino	28	3817	
Upregulated in surface	7	4204	0.0001
Upregulated in Pachón	70	3797	
Upregulated in surface	34	4038	0.0002
Upregulated in Tinaja	47	4653	
Upregulated in surface	10	5643	< 0.00001

Table K. *A. mexicanus* orthologs of genes that are light induced in zebrafish are not more often upregulated in cavefish compared to surface fish. *P*-values for each surface-cave comparison produced with a hypergeometric test.

	Upregulated light-activated genes	Upregulated genes	Significance
Tinaja	49	4700	$p = 0.85$
Surface	70	5653	
Pachón	46	3867	
Surface	66	4072	$p = 0.96$
Molino	37	3845	
Surface	63	4211	$p = 0.99$

Fig A. Raw reads per sample for Molino.

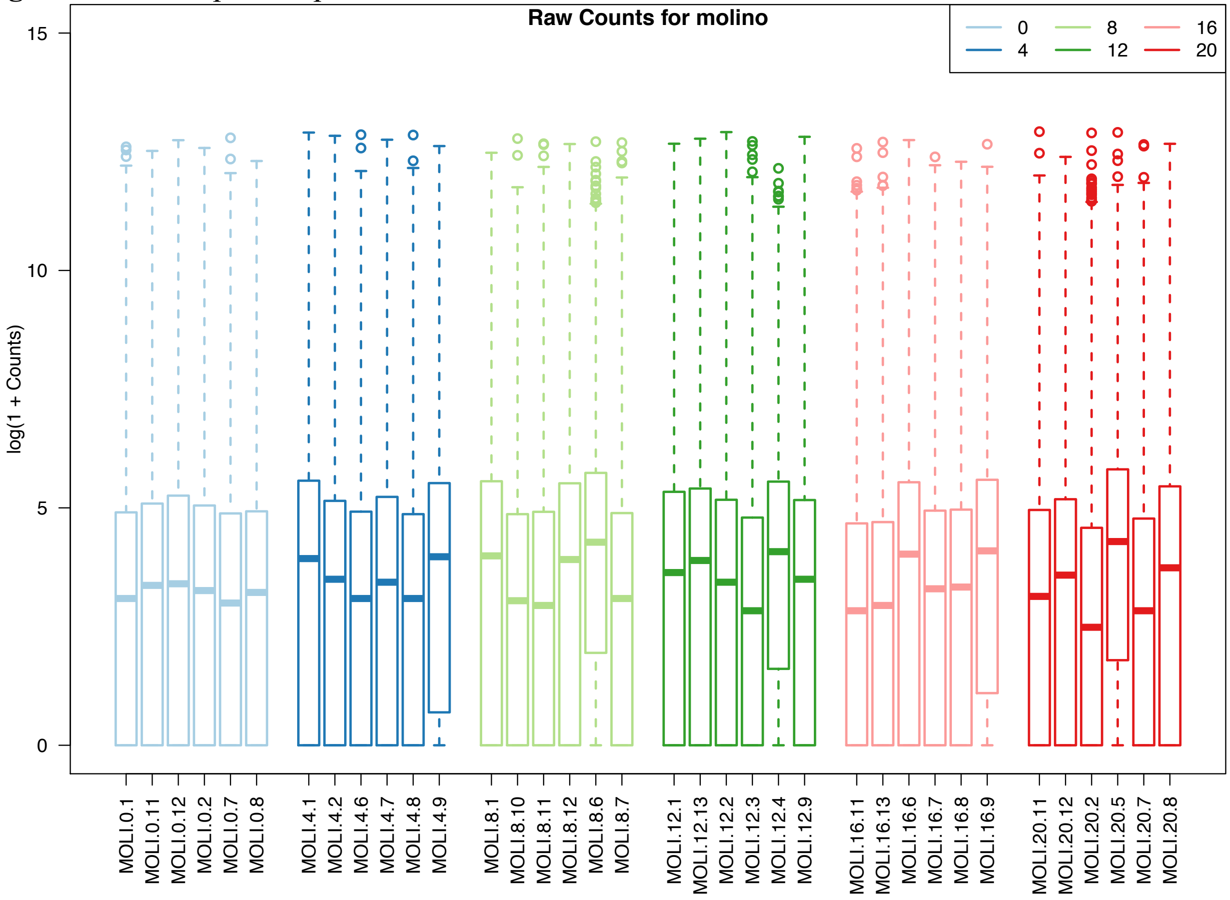

Fig B. Raw reads per sample for Pachón.

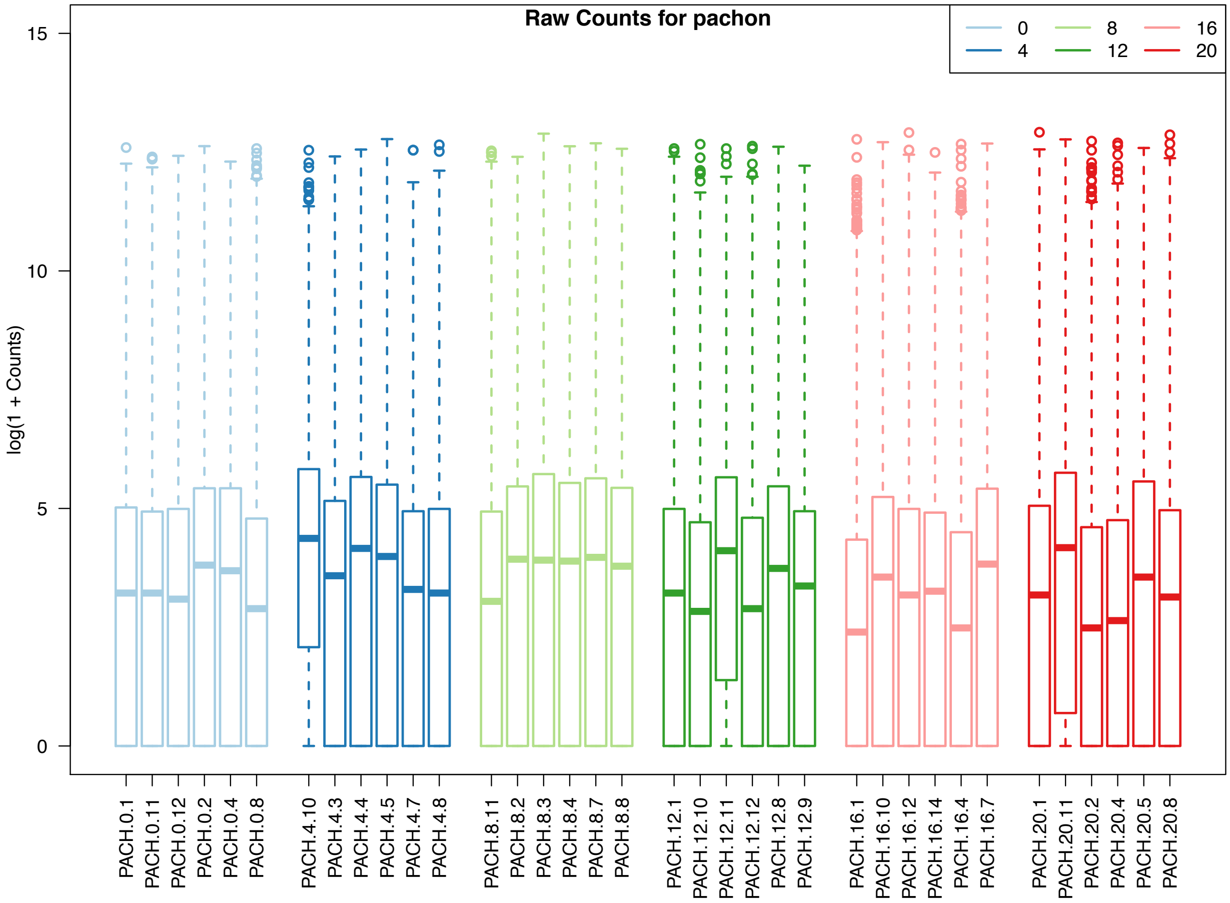

Fig C. Raw reads per sample for surface fish.

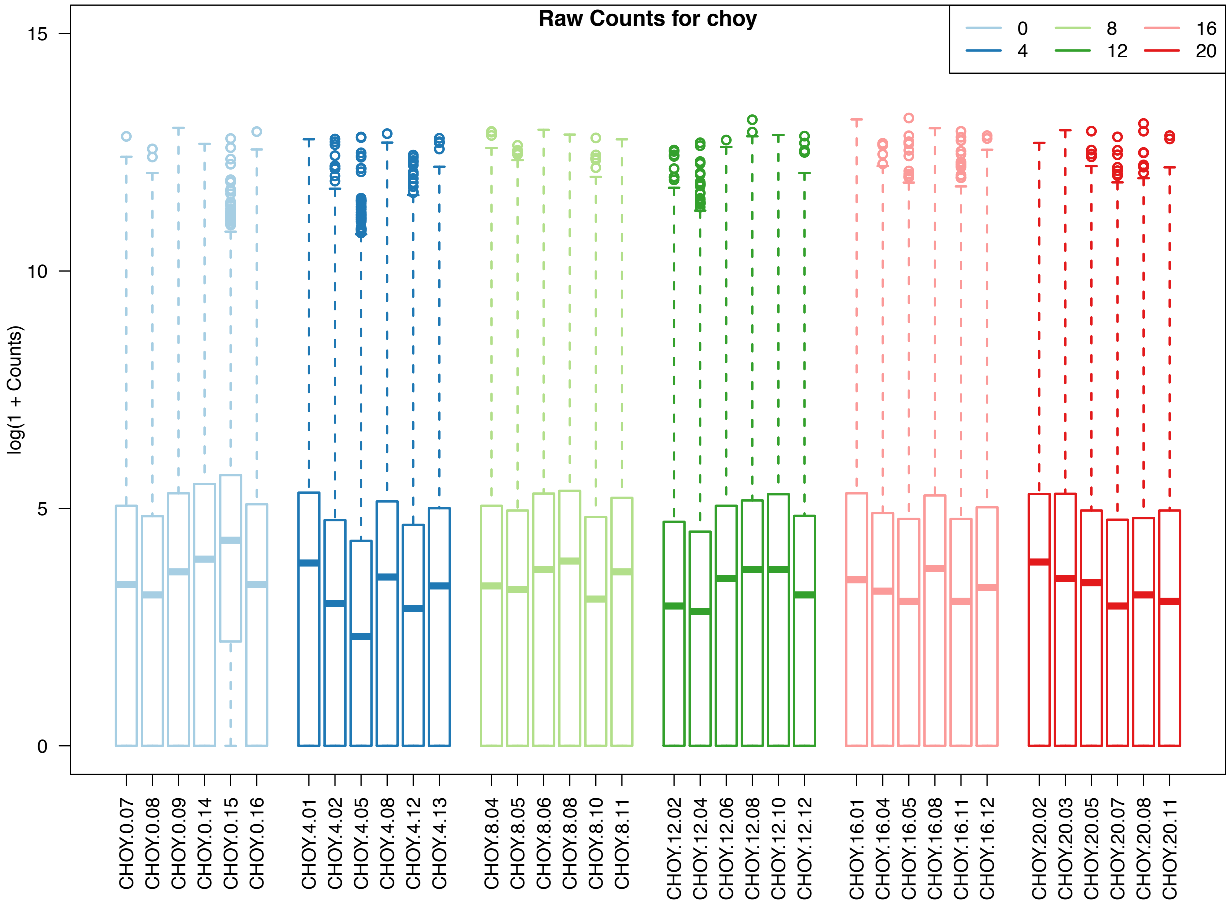

Fig D. Raw reads per sample for Tinaja.

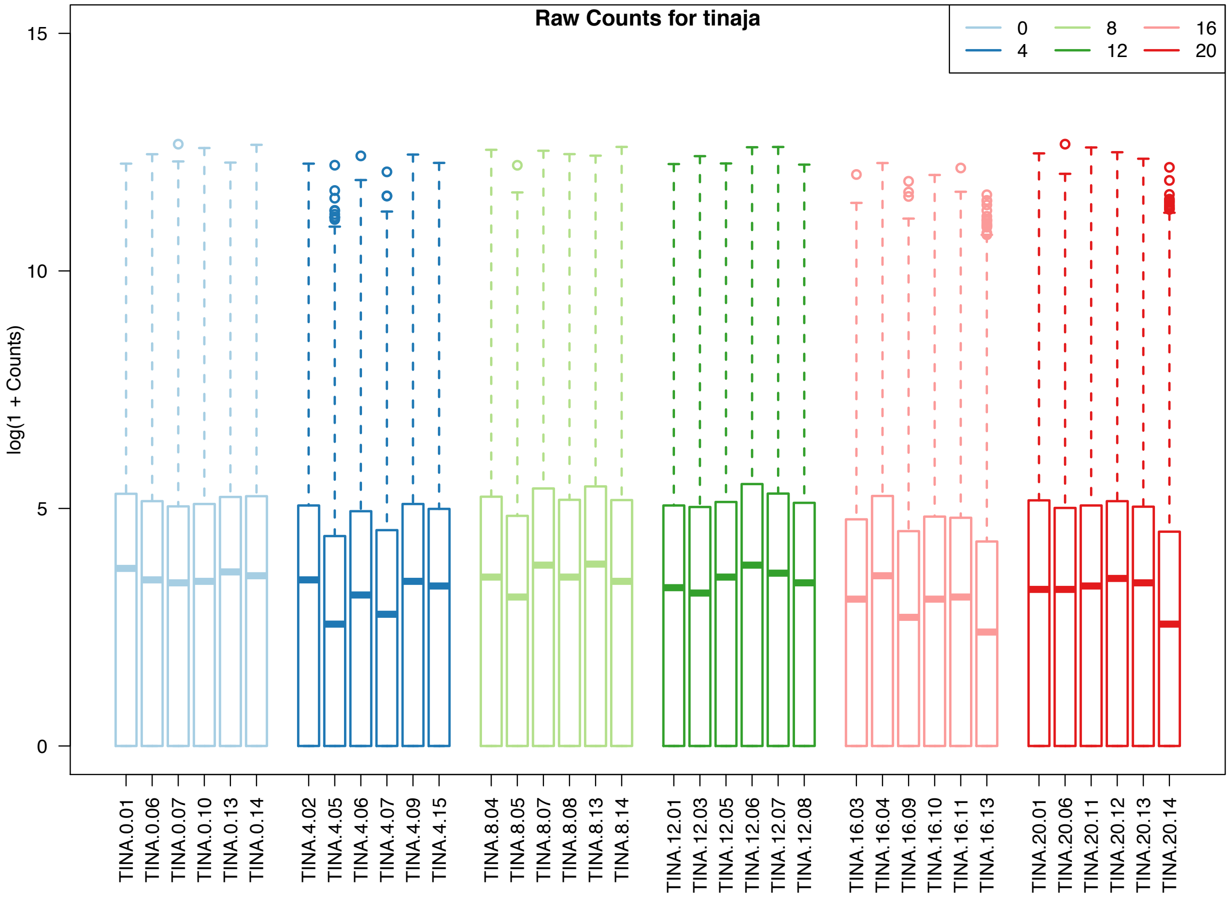

Fig E. A. PC1 and PC2 (explaining 19.1% and 18% of variation, respectively) show that the primary axes of differentiation among samples is ecotype. B. PC3 (explaining 7.1% of variation) separates Molino from other populations.

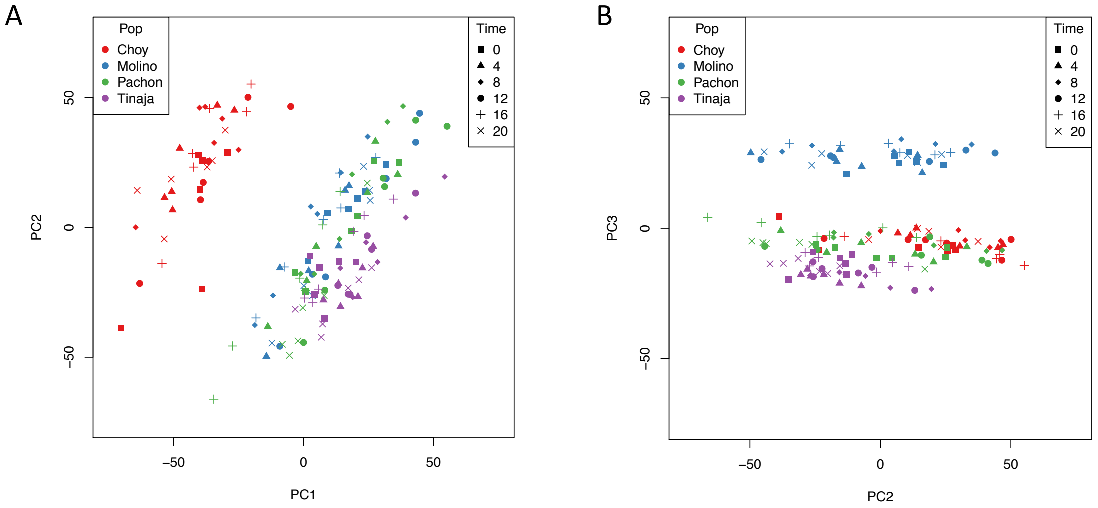

Fig F. Cave populations show shifts in phase at *per1a/b* and *cry1a, w*ith gene expression peaking later in cave populations compared to the surface population. Expression is represented as normalized read counts.

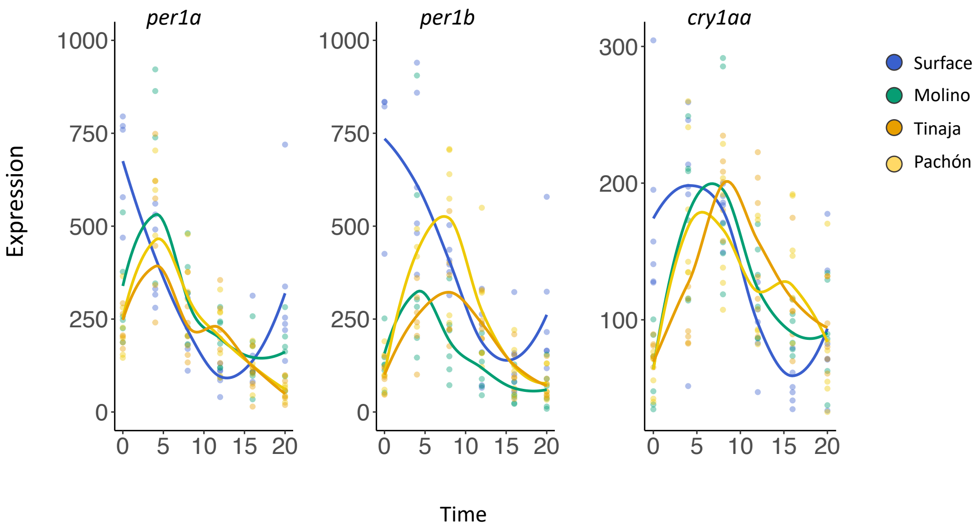

Fig G. A-B. In the circles are the circadian phase distributions of predicted targets of RRE and E-BOX for surface fish. Grey bars represent the proportion of each motif seen in each phase. Highlighted in light grey are intervals of phase-specific enrichment for surface fish for each motif. Genes with the RRE motif (C) and EBOX (D) motifs show shifts in the timing of peak expression in cavefish populations.

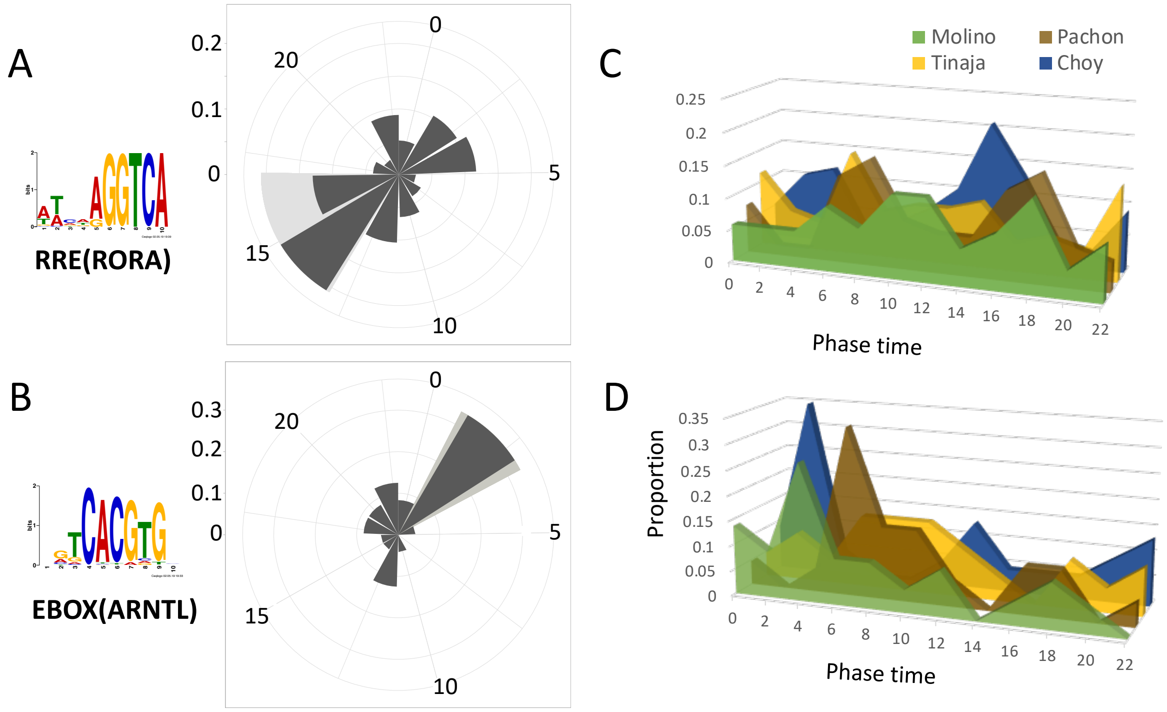

Fig H. In the circles are the circadian phase distributions of predicted targets of the D-Box (NFIL3) for surface fish. Grey bars represent the proportion of motifs seen in each phase. Highlighted in light grey is the interval with most significant phase-specific enrichment for surface fish.

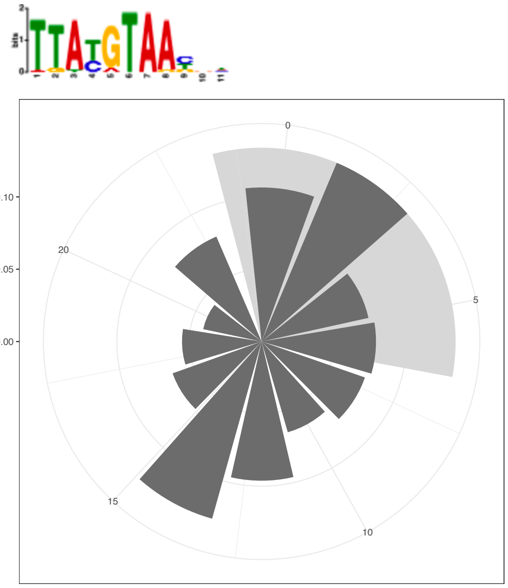

Fig I. Region of interest used in all brain images (white box). Regions include the optic tectum (TeO) and periglomerular grey zone (PGZ) shown in relation to coronal section stained with DAPI. Scale bar is 50μM.

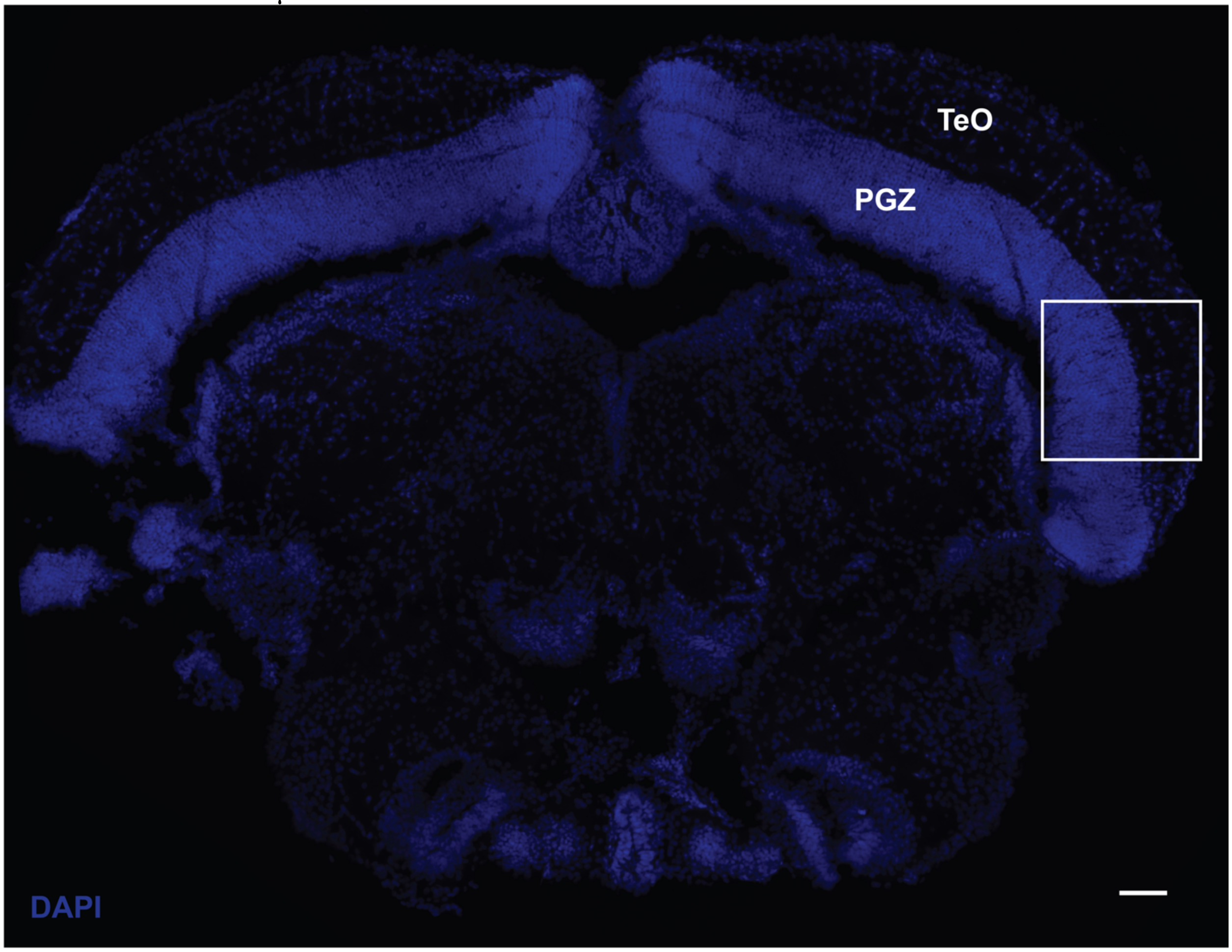

Fig J. DAPI staining in brain ('B', top panels for each timepoint) and liver ('L', bottom panels for each timepoint) of surface fish and cavefish (Pachón, Tinaja, Molino) at CT0, CT8, and CT16. A. DAPI channel for sections included in Fig 4A. B. DAPI channels for sections included 4B.

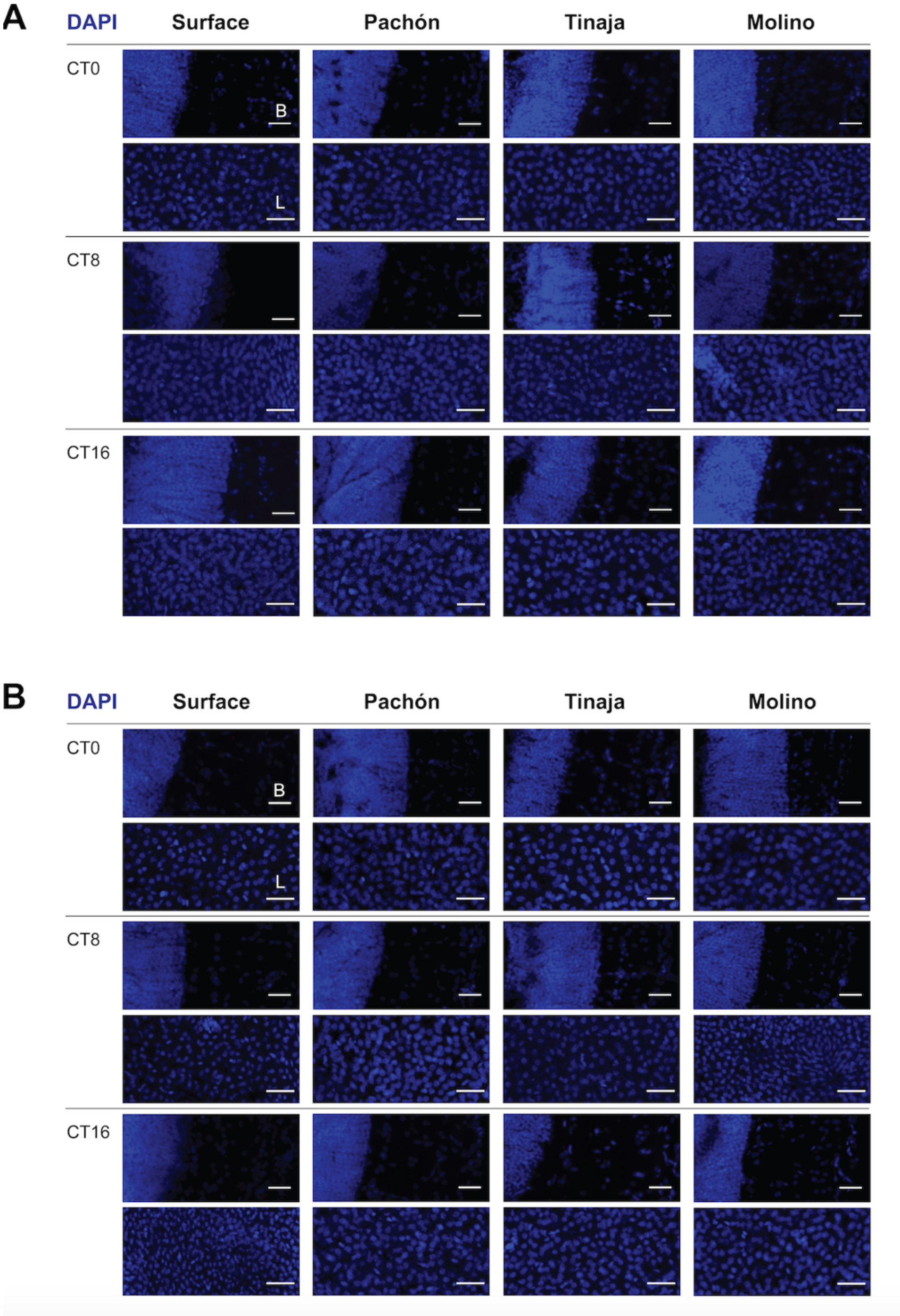

Fig K. Expression patterns of *per1a* and *arntl1a* in Tinaja and Molino brain images adjusted to correct for oversaturation.

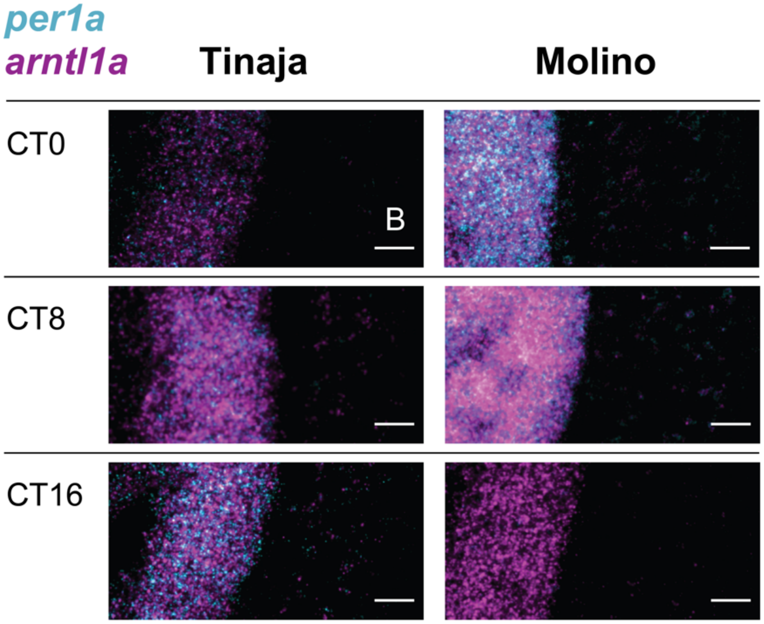

Fig L. Temporal expression patterns of (A) *per1a* and (B) *arntl1a* in midbrain and liver tissue in *Astyanax mexicanus* populations. *In-situ* staining of *rorca* (A) and *rorcb* (B) using RNAscope® in midbrain ('B', top panels for each timepoint) and liver ('L', bottom panels for each timepoint) of Surface fish and cavefish (Pachón, Tinaja, Molino) at CT0, CT8, and CT16. Each time point is a single fish sample. Images are representative sections of two fish collected per time point, per population. Scale bar is 25μM.

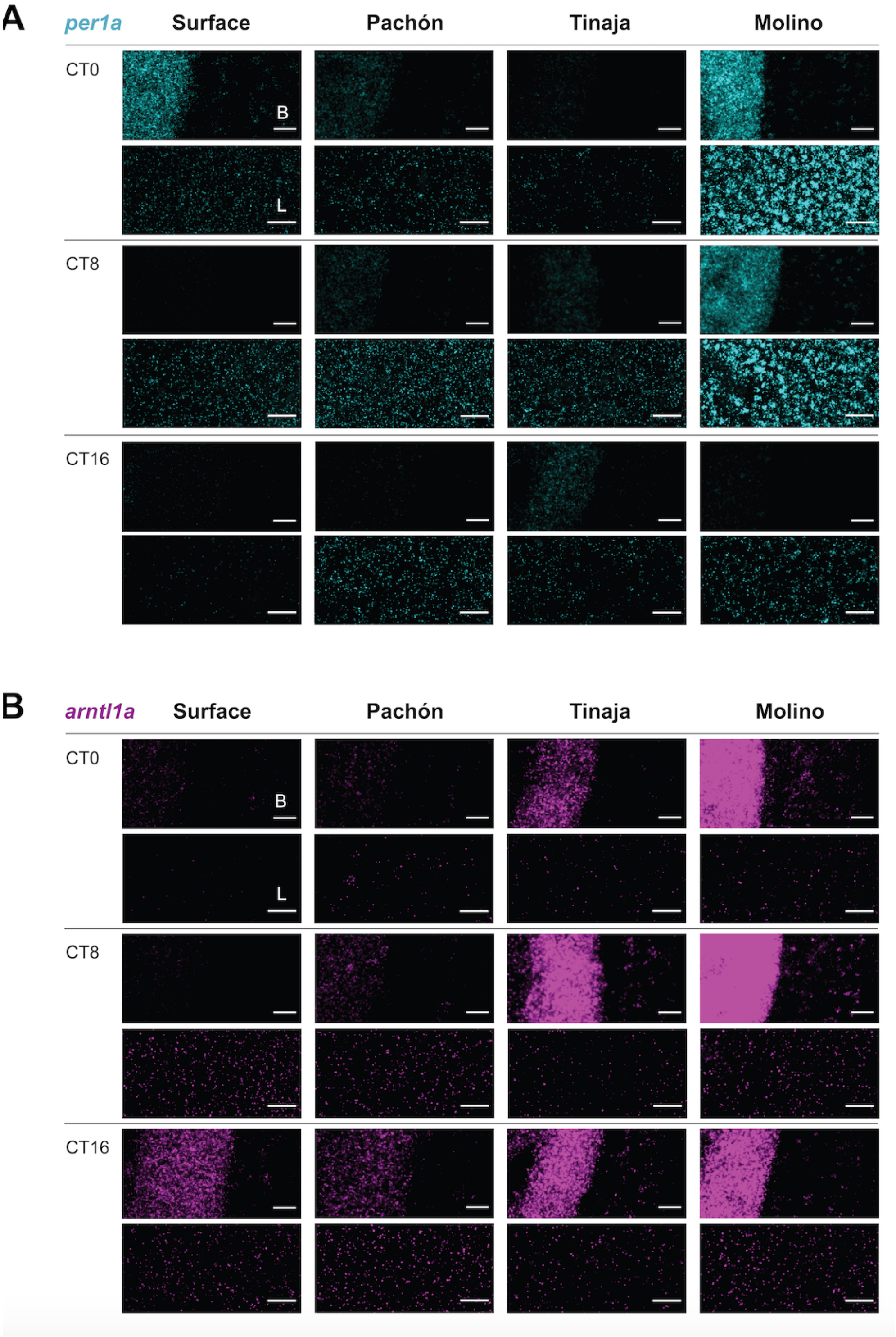

Fig M. Temporal expression patterns of (A) *rorca* and (B) *rorcb* in midbrain and liver tissue in *Astyanax mexicanus* populations. *In-situ* staining of *rorca* (A) and *rorcb* (B) using RNAscope® in midbrain ('B', top panels for each timepoint) and liver ('L', bottom panels for each timepoint) of Surface fish and cavefish (Pachón, Tinaja, Molino) at CT0, CT8, and CT16. Each time point is a single fish sample. Images are representative sections of two fish collected per time point, per population. Scale bar is 25µM.

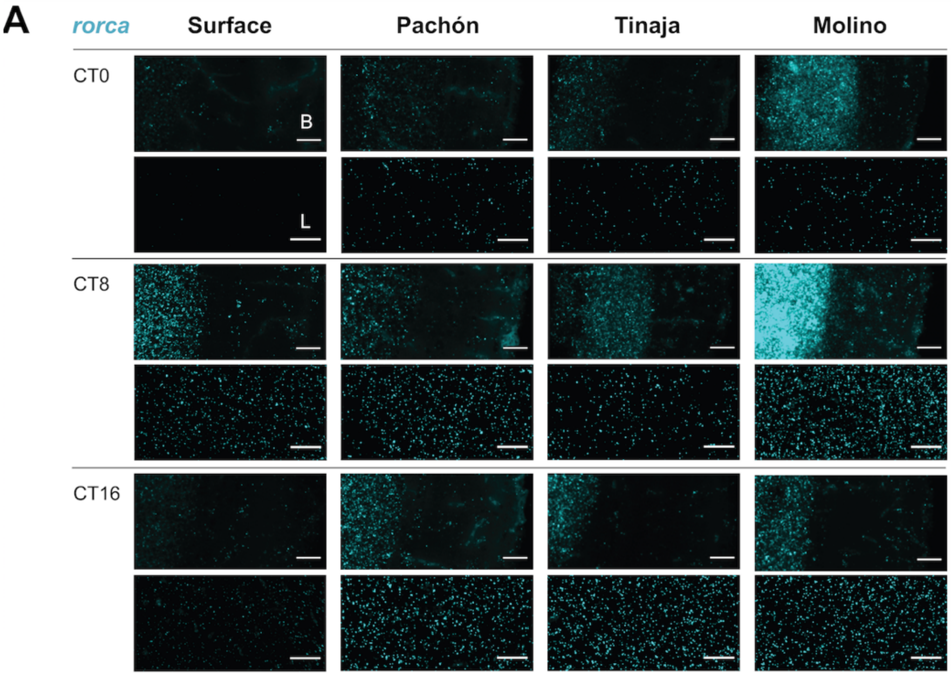

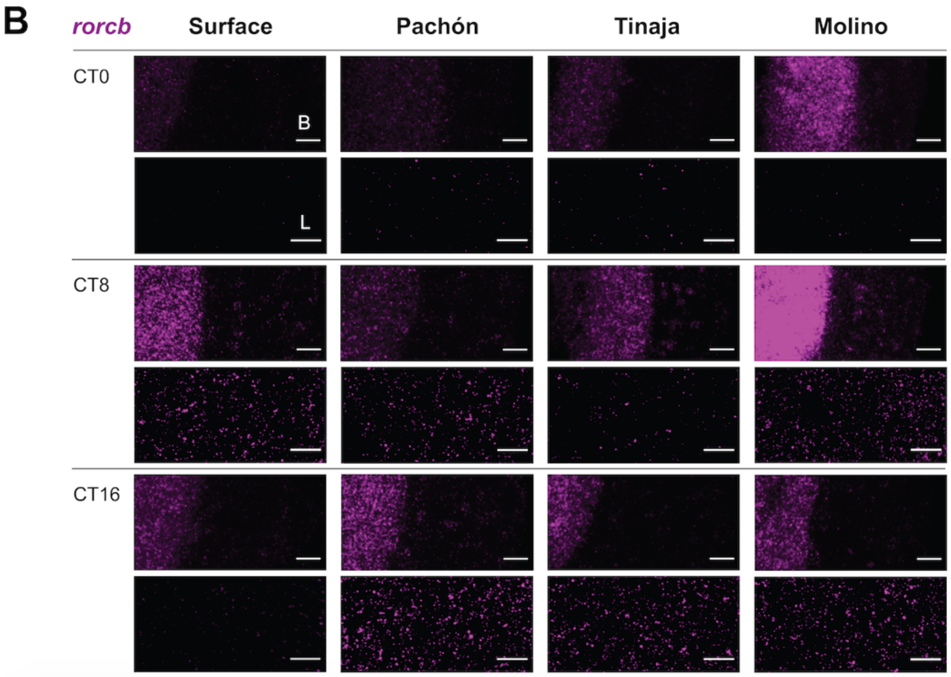

Fig N. *Per1a* expression at each time point measured with RNAseq in whole fry and in the brain and liver with RNA FISH. Grey dotted lines represent a loess regression for visualization purposes.

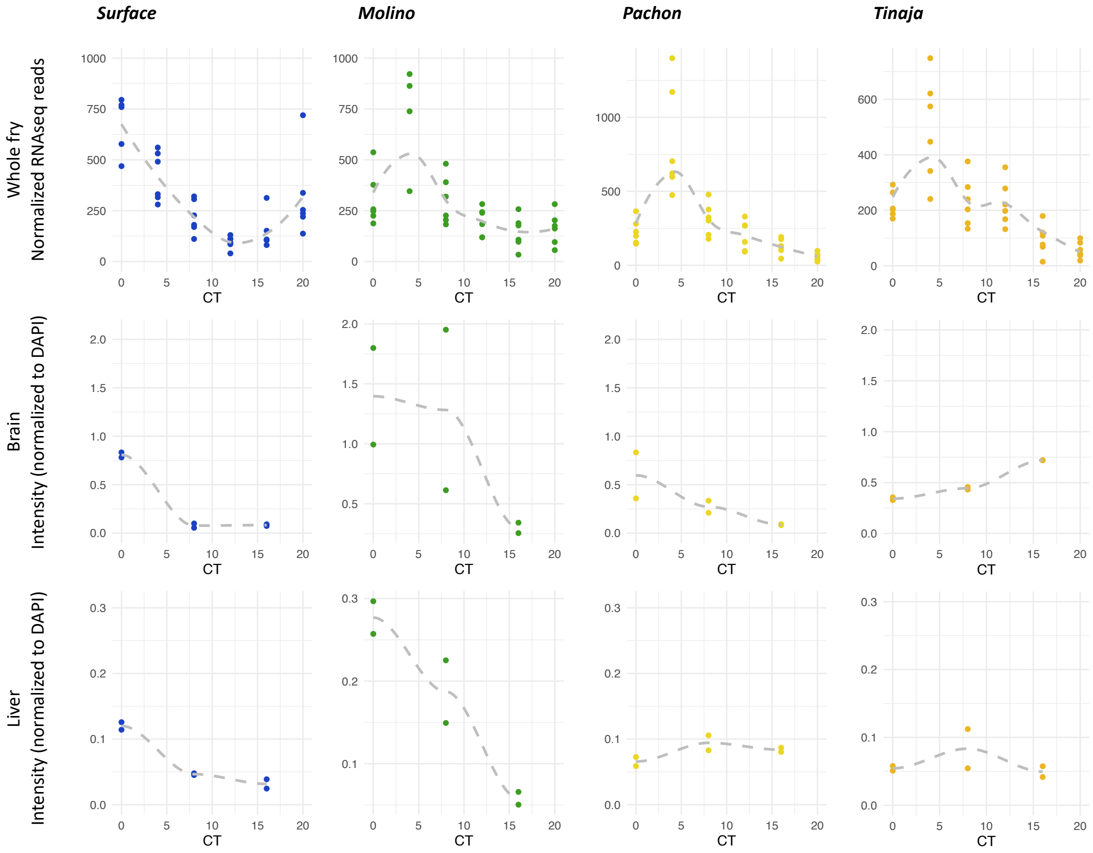

Fig O. *Arntl1a* expression at each time point measured with RNAseq in whole fry and in the brain and liver with RNA FISH. Grey dotted lines represent a loess regression for visualization purposes.

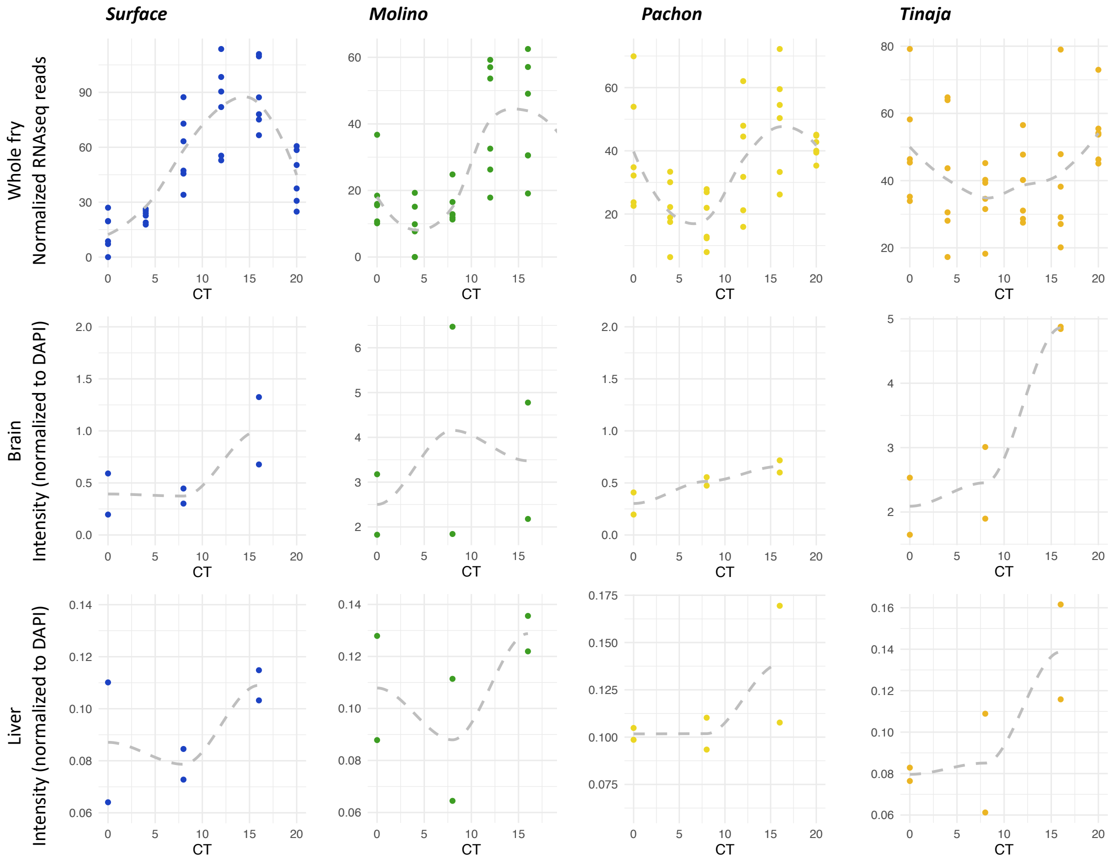

Fig P. *Rorca* expression at each time point measured with RNAseq in whole fry and in the brain and liver with RNA FISH. Grey dotted lines represent a loess regression for visualization purposes.

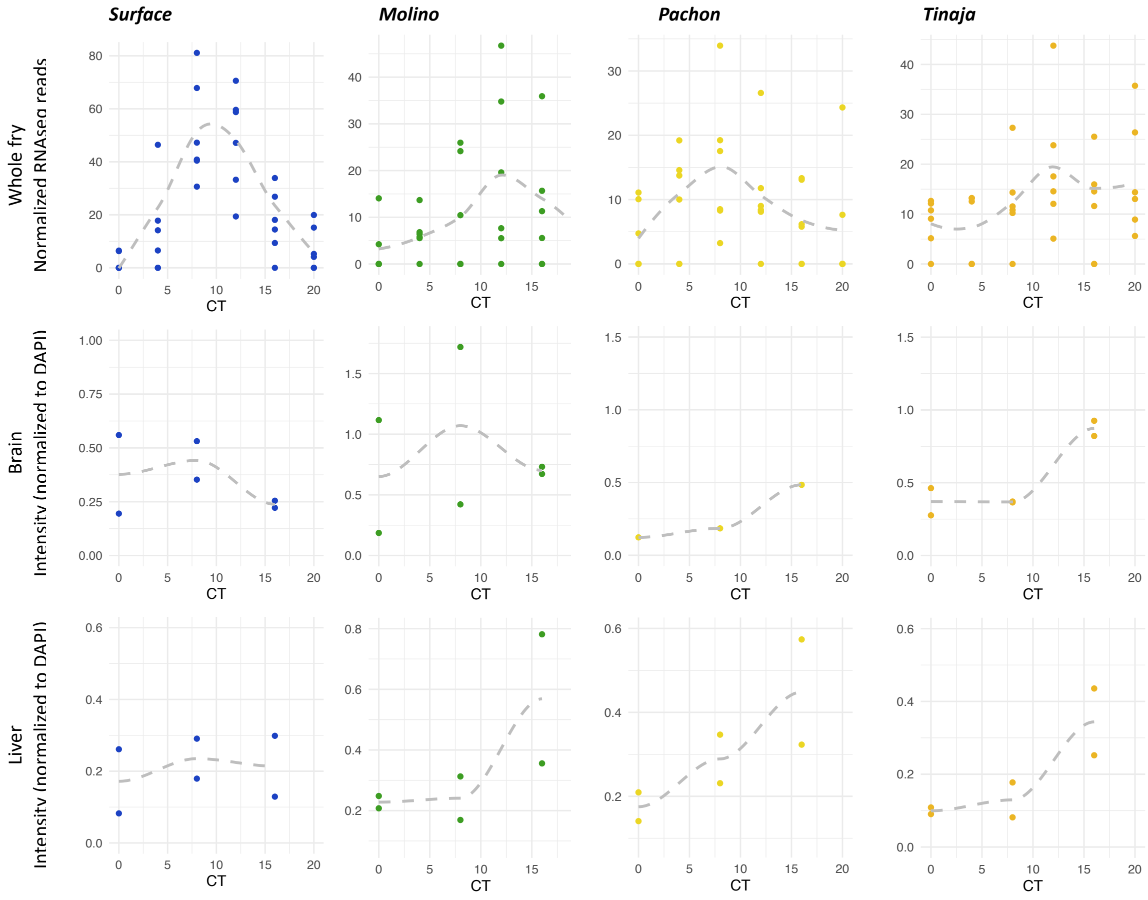

Fig Q. *Rorcb* expression at each time point measured with RNAseq in whole fry and in the brain and liver with RNA FISH. Grey dotted lines represent a loess regression for visualization purposes.

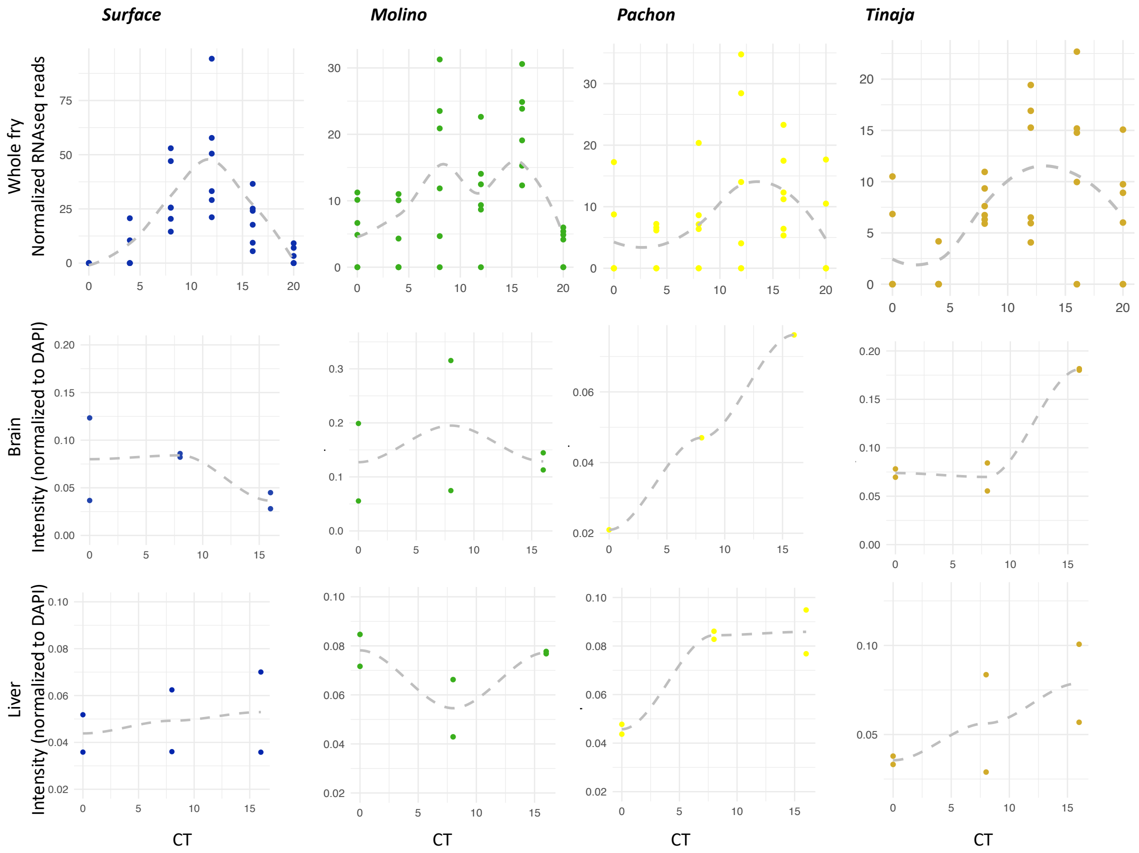

Fig R. Analysis of mutagenesis in *aanat2* crispant F_0 fish. **A**. Genotyping gel of uninjected control and injected embryos. A portion of *aanat2* genomic region was amplified by PCR from DNA extracted from individual embryos. Labeled D is half of the PCR product that was digested with BbvI. Unlabeled is undigested PCR product. Indels can disrupt the restriction enzyme site, leading to undigested PCR product in injected embryos. **B**. Diagram of *aanat2* gene based on the surface fish reference genome (Ensembl v98). Boxes indicate exon and lines indicate introns. The empty boxes are 5' and 3' UTR and the closed boxes are coding sequence. A gRNA was designed targeting exon 1. The gRNA target site is in blue and the PAM sequence is in red. The underlined sequence is the BbvI restriction enzyme recognition sequence used for genotyping. The arrow indicates the predicted Cas9 cut site. Gene structure was generated using http://wormweb.org/exonintron and then modified. **C**. Sequence of wildtype surface fish and sequence of six clones from the restriction enzyme resistant band from *aanat2* injected individuals. The total number of base pairs less than the wildtype sequence is indicated to the right of each clone.

A

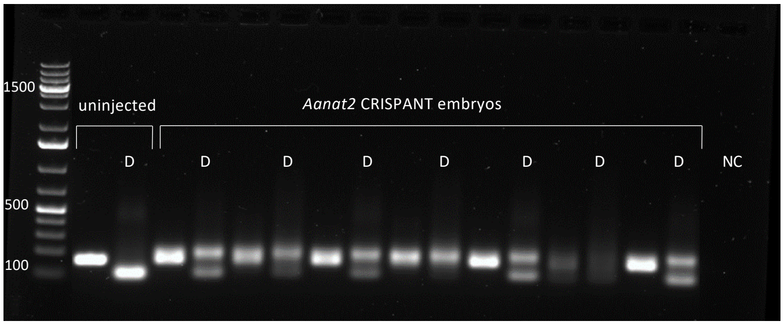

B

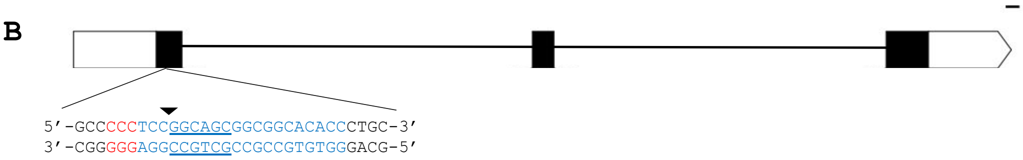

C

```
CCAGCCCCCTCCGGCAGCGGCGGCACACCCTGCCCGCCAGCGAGTTCCGCAACCTCACGCCGCAGGACGCCATCAGCGTGTTCGAG   WT
CCAGCCC----------------------------GCCAGCGAGTTCCGCAACCTCACGCCGCAGGACGCCATCAGCGTGTTCGAG   -28
CCAGCCCCCTCCAGCGAGC-------------------------TCCGCAACCTCACGCCGCAGGACGCCATCAGCGTGTTCGAG   -23
CCAGCCCCCTCAGCG—GCGGCGGCACACCCTGCCCGCCAGCGAGTTCCGCAACCTCACGCCGCAGGACGCCATCAGCGTGTTCGAG   -1
CCAGCCCCCTCCAGCCCC-----CACACCCTGCCCGCCAGCGAGTTCCGCAACCTCACGCCGCAGGACGCCATCAGCGTGTTCGAG   -5
CCAG-----------------GGCACACCCTGCCCGCCAGCGAGTTCCGCAACCTCACGCCGCAGGACGCCATCAGCGTGTTCGAG   -17
CCAGCCCC-----CAGCGGCGGCACACCCTGCCCGCCAGCGAGTTCCGCAACCTCACGCCGCAGGACGCCATCAGCGTGTTCGAG   -5
```

Fig S. Analysis of mutagenesis in *rorca* crispant F_0 fish. **A**. Genotyping gel of uninjected control and injected embryos. A portion of *rorca* genomic region was amplified by PCR from DNA extracted from individual embryos. Labeled D is half of the PCR product that was digested with Cac8I. **B**. Diagram of *rorca* gene based on the Pachón Ensembl v93 genome. Boxes indicate exon and lines indicate introns. The empty boxes are UTR and the closed boxes are coding sequence. A gRNA was designed targeting exon 6. The gRNA target site is in blue and the PAM site is in red. The underlined sequence is the Cac8I restriction enzyme recognition sequence used for genotyping. The arrow indicates the predicted Cas9 cut site. Gene structure was generated using http://wormweb.org/exonintron and then modified. **C**. Sequence of wildtype surface fish and sequence of 3 clones from the restriction enzyme resistant band from *rorca* injected individuals. The total number of base pairs more or less than the wildtype sequence is indicated to the right of each clone.

A

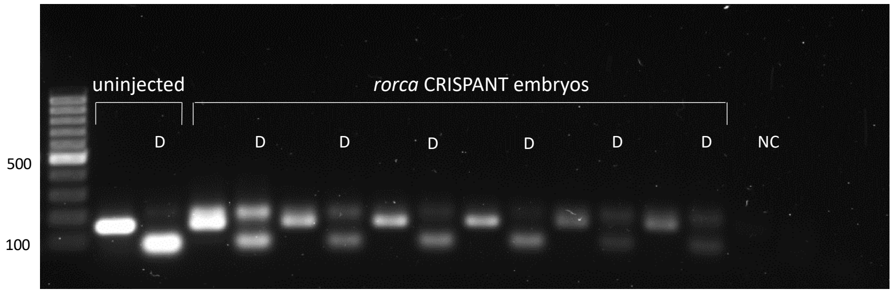

B

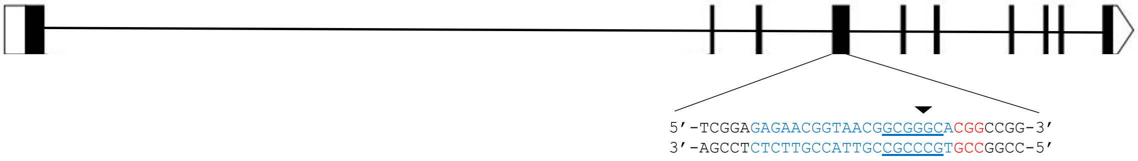

C

```
GACATCGGAGAGAACGGTAACGGCGGGCACGGCCGGGCGTACAGCCGCAGCAGCTCCAGCACGGCCCTCAGCGACCTGGACGAC   WT
GACATCGGAGAGAACGGTAAC------------------------CG----AGCTCCAGCACGGCCCTCAGCGACCTGGACGAC  -28
GACATCGGAGAGAACGGTAACGGCGGGCGTAACGGCCGGGCGTACAGCCGCAGCAGCTCCAGCACGGCCCTCAGCGACCTGGAC   +3
GACATCGGAGAGAACGGTAACCGC------------------------AGCAGCTCCAGCACGGCCCTCAGCGACCTGGACGAC  -22
```

Fig T. A. Expression of *per2*, a light-activated clock gene, over the course of the day in surface and cave populations (JTK_cycle *p*-values: surface, p=0.09, q=0.7; Molino, p=0.07, q=1; Pachón, p=0.003; q=0.16; Tinaja, p=0.016; q=0.22). Rhythmicity was not found to be different between populations (all comparisons, S_{DR} p>0.68, q=1). **B.** Base level expression of *per2* between populations. *Per2* has lower base level expression in the surface than Pachón (log_2-fold change=0.82) and Tinaja (log_2-fold change=0.56), but higher expression than Molino (log_2-fold change=1.3).

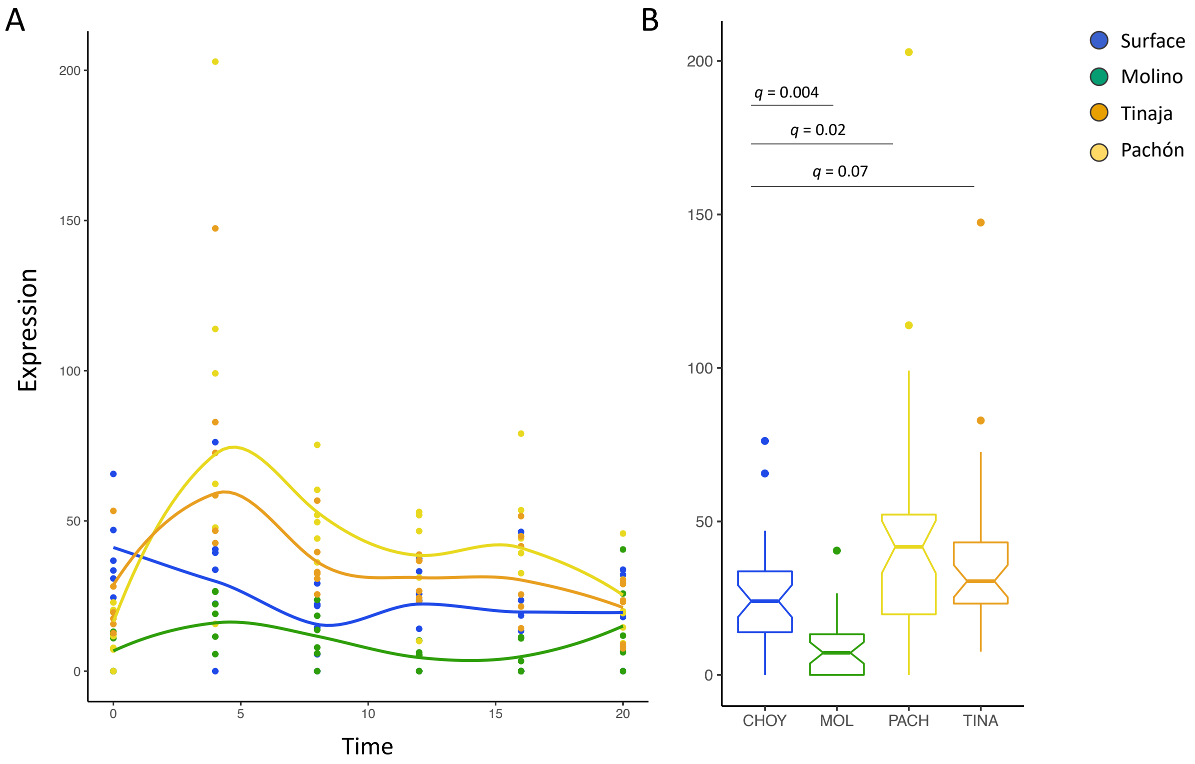

Fig U. Core circadian genes and melatonin regulator *aanat2* show differentiated rhythmicity from surface fish in at least one cave population.

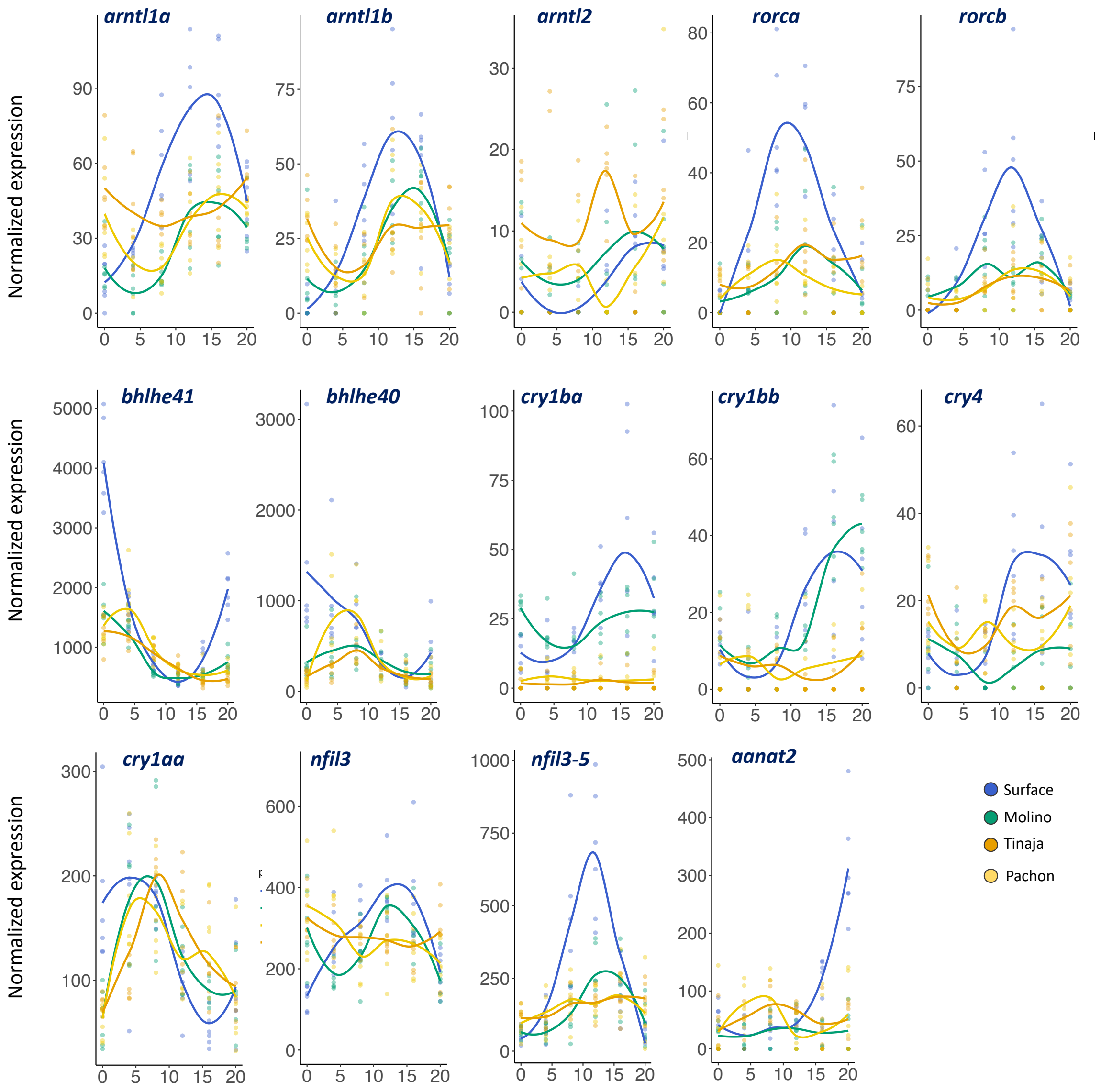

Fig V. *Exo-rhodopsin* has robust rhythmic expression in the surface population (p=9.26 x 10^{-5}, q = 0.006), but is not strongly in rhythmic in cave populations (Pachón, p=0.04, q = 0.32; Tinaja, p=1, q = 1.0; Molino, p=0.37, q = 1.0).

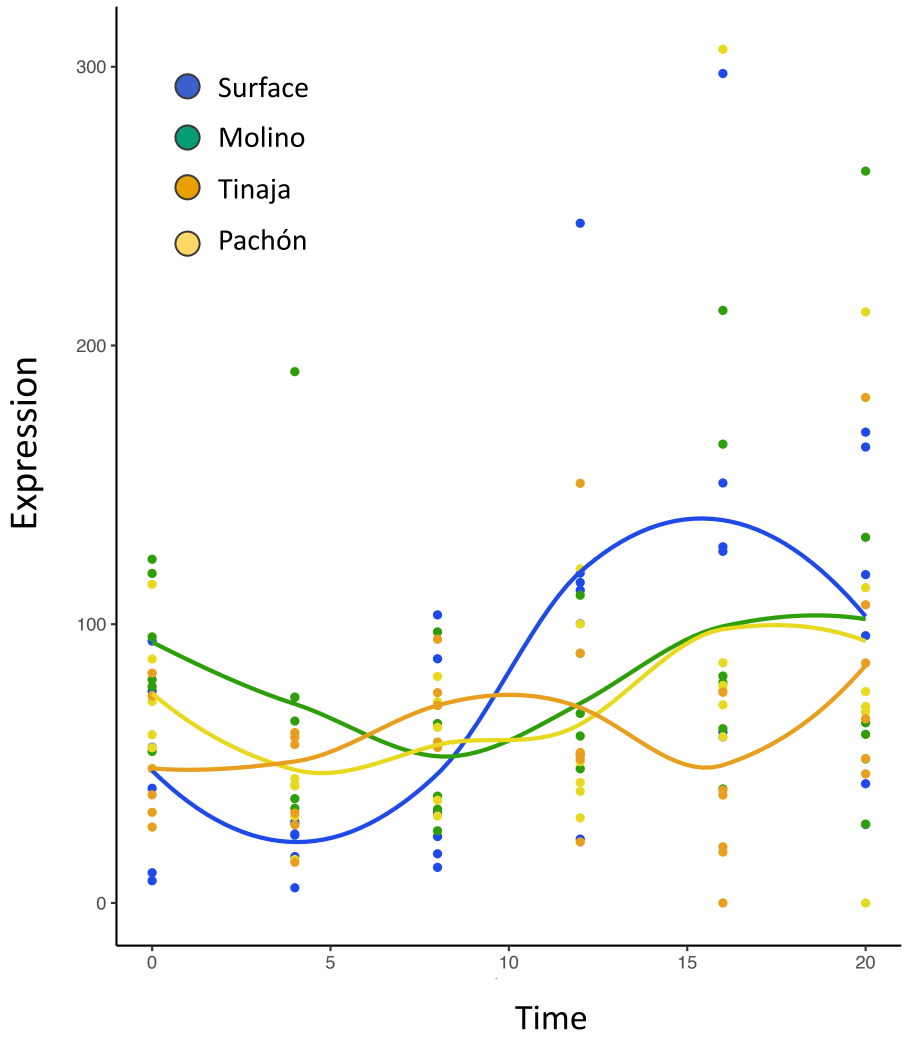

Fig W. Phylogenetic tree of species used for RELAX analysis for changes in selection intensity. Black branches indicate "reference" branches, where blue branches (cavefish lineages of *A. mexicanus*) have been used as foreground branches to test for changes in selection intensity.

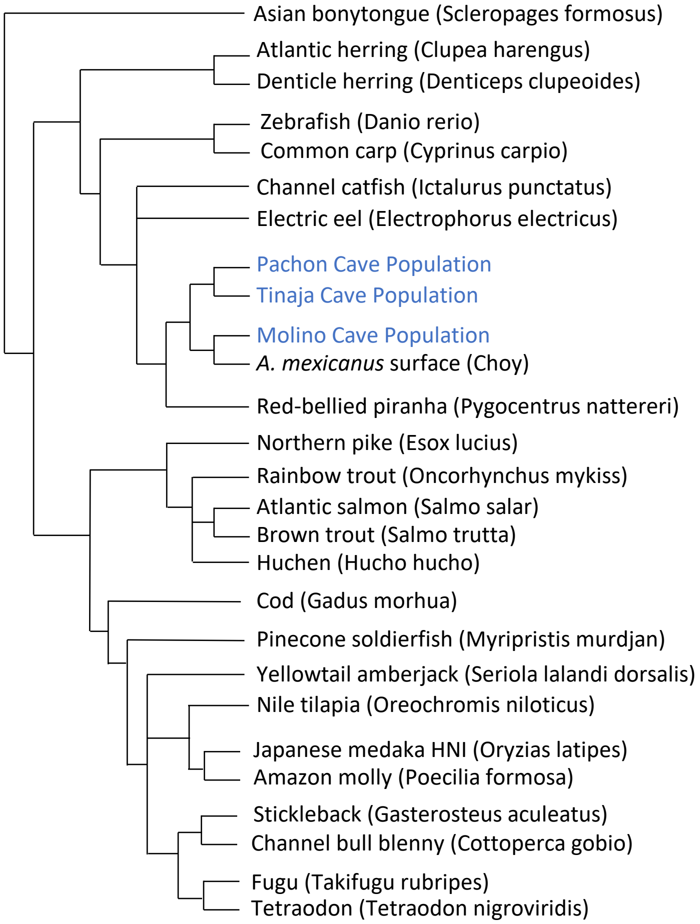

Supplement: S1 Text — Table A. Numbers of rhythmic genes in each population. Table B. Number of genes with loss in rhythmicity (P > 0.5) in cave populations compared to rhythmic expression in surface (FDR < 0.1 and FDR < 0.05). Table C. Known circadian regulators that are arrhythmic in one or more cave populations. Table D. Timing of peak expression of core clock genes (primary and accessory loops) compared between zebrafish and A. mexicanus populations. Table E. Phase shifts between surface and cave populations. Table F. The number of significant circadian binding motifs identified in promoter proximal regions of genes with evidence for transcription (p<0.05) in the surface population. Table G. Arrhythmic genes in cave populations where motif sequences are also lost. Table H. Average timing difference in peak expression of circadian feedback loop targets. Table I. Number of genes with a significant differential rhythmicity score. Table J. Genes associated with GO term DNA-repair are upregulated in cave populations more than expected by chance. P-values based on Fisher’s exact tests. Table K. A. mexicanus orthologs of genes that are light induced in zebrafish are not more often upregulated in cavefish compared to surface fish. P-values for each surface-cave comparison produced with a hypergeometric test. Fig A. Raw reads per sample for Molino. Fig B. Raw reads per sample for Pachón. Fig C. Raw reads per sample for surface fish. Fig D. Raw reads per sample for Tinaja. Fig E. A. PC1 and PC2 (explaining 19.1% and 18% of variation, respectively) show that the primary axes of differentiation among samples is ecotype. B. PC3 (explaining 7.1% of variation) separates Molino from other populations. Fig F. Cave populations show shifts in phase at per1a/b and cry1a, with gene expression peaking later in cave populations compared to the surface population. Expression is represented as normalized read counts. Fig G. A-B. In the circles are the circadian phase distributions of predicted targets of RRE [file pgen.1009642.s001.pdf]
